# Comparative effects of raw and processed cistanche glycosides on the HPT axis and gut microbiota in a rat model of kidney-yang deficiency

**DOI:** 10.3389/fphar.2025.1597564

**Published:** 2025-07-25

**Authors:** Xiaoqing Shen, Jing Lian, Chao Zhang, Yixiang Miu, Yuan Zhang, Ji Shi, Nan Xu, Tianzhu Jia

**Affiliations:** ^1^ Pharmaceutic Department, Liaoning University of Traditional Chinese Medicine, Dalian, Liaoning, China; ^2^ Key Research Laboratory of Traditional Chinese Medicine Processing Process Principles of the State Administration of Traditional Chinese Medicine, Dalian, China; ^3^ Liaoning Provincial Traditional Chinese Medicine Processing Professional Technology Innovation Center, Dalian, China

**Keywords:** *Cistanche deserticola*, feces metabolomics, gut microbiota, total glycosides, HPT axis

## Abstract

**Introduction:**

Kidney Yang Deficiency (KYD) is a metabolic disorder associated with kidney damage. Its slow progression means that causative factors and effective therapeutic agents remain unclear. Extensive evidence links KYD to gut microbiome metabolic diseases and the Hypothalamic-Pituitary-Thyroid (HPT) axis. *Cistanche deserticola* (CD) is a commonly used traditional Chinese medicine for treating KYD. However, the precise interactions between gut microbiota and KYD, as well as the mechanisms of raw and processed CD total glycosides (CDG) in modulating KYD, require further investigation. This study aims to evaluate the effects and mechanisms of CDG in a KYD rat model using 16S rRNA gene sequencing and fecal metabolomics.

**Methods:**

CDG was extracted from both raw and processed CD and analyzed via HPLC. Propylthiouracil-induced KYD rats were used to assess pharmacological effects, including serum levels of T_3_, T_4_, TSH, TRH, FFA, LPL, and NO; organ indices of the spleen, kidney, and thymus; blood cAMP/cGMP levels; and liver levels of glycogen, SDH, Ca^2+^-ATPase, and Na^+^-K^+^-ATPase. Immunohistochemistry was also performed.

**Results:**

Fecal non-targeted metabolomics identified 98 metabolites associated with KYD, while 16S rRNA sequencing revealed 13 key intestinal microbiotas linked to KYD. CDG therapy effectively alleviated KYD symptoms by modulating the gut microbiota, improving metabolic and microbial imbalances in KYD. RG/WG significantly improves KYD rats mainly through the relationship between the intestinal microbiota and arachidonic acid metabolism. The key bacterial genera *lleibacterium* and *Streptococcus* observed in the changes of intestinal flora and fecal metabolite content were significantly negatively correlated with phosphatidylcholine and phosphatidylethanolamine.

**Discussion:**

This integrative approach of gut microbiome and fecal metabolomics not only provides a scientific basis for CDG’s preventive effects on KYD via the HPT axis but also elucidates the potential mechanisms underlying CDG’s action against KYD.

## Graphical Abstract

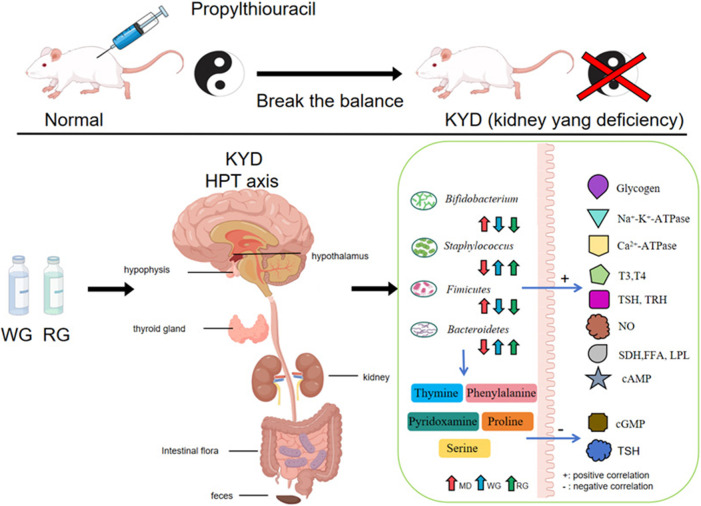

## 1 Introduction

KYD in the contemporary TCM clinic (Zhou et al., 2024), is a type of condition that results from kidney-Yang insufficiency. Feeling powerless and early ejaculation, urinary tract infections, urinary frequency, weakness and discomfort in the knees and waist are the most common signs (Zhang M-L. et al., 2024). According to pathological research, the HPT dysfunction was the primary source of the aberrant symptoms of KYD (Zhang X-Y. et al., 2024; Iglesias and Díez, 2009). In addition, it is also found that this disease is transmitted through the downward link of the neuroendocrine immune (NEI) network, which adversely feeds back to the seek glands, such as the pituitary and hypothalamus, creating a spontaneous control loop of the hypothalamus-pituitary-thyroid axis (Ayala et al., 2011). At present, KYD animal models induced by hydrocortisone (Sun et al., 2023) and propylthiouracil (Zheng et al., 2024) have been established. The 2020 Chinese Pharmacopoeia includes *Cistanche deserticola* Y .C.Ma and *Cistanche tubulosa* (Schenk) Wight (Pharmacopeia of the People’s Republic of China, 2020). Among these varieties, *Cistanche deserticola* Y. C. Ma (*Roucongrong* in Chinese) is a dry, scaly fleshy stem of the Orobanchaceae plant *Cistanche deserticola*. CD has been paid more and more attention by pharmacologists because of its natural safety and significant role in preventive healthcare. According to TCM (Zhang X. et al., 2024), the raw products can moisten the intestines and purge them, while the processed Cistanche has a stronger effect of tonifying the kidney, strengthening yang, and strengthening tendons and bones (Yin et al., 2023). In 2021, the People’s Republic of China’s National Health and Welfare Commission announced this research object of *medicine and food homologous* medicinal materials (Zhou et al., 2023). At present, CD and wine-processed CD have increasingly become the main monarch drug in the compounds of modern Chinese medicine clinical treatment of KYD and infertility (Li Z. et al., 2024). Although the treatment of KYD and its related symptoms has a long history in TCM, from the perspective of modern science, especially in the context of the HPT axis. Comparative studies on the improvement of kidney-tonifying and yang-enhancing functions in KYD model rats are still relatively few. Our previous research compared the chemical constituents of CDG using UPLC-Q-TOF-MS^E^ (Li et al., 2021). However, these studies mainly focus on the qualitative and semi-quantitative analysis of the components. But there is still a lack of sufficient evidence on how these main chemical constituents are directly related to the efficacy of the botanical drug.

In this study, CD and wine-processed CD were used as the research objects, and the differences between them were comprehensively evaluated from the aspects of plant chemical composition, impacts of drugs, bioactive molecules, and potential mechanisms of action. The chemical substances of traditional Chinese medicine before and after processing are similar, the content is significantly different, and the two kinds of traditional Chinese medicine decoction pieces show different pharmacological effects on KYD rats induced by propylthiouracil. Thus, by combining the metabolic exposure of the main metabolites in feces with their regulatory function on the microbiota of the gut and feces metabolites *in vivo*, a combined effect index (CEI) was created to assess the effectiveness of metabolites. Furthermore, the pharmacological benefits and safety of CD’s raw and processed products in enhancing KYD syndrome and energy metabolism disorders were further described in conjunction with H&E staining, immunohistochemistry and PCR analysis (Guo et al., 2019), and the potential pharmacodynamic mechanism was revealed. At the same time, it also proved the necessity of CD as a kidney-yang medicine. Additionally, their contents of 6 main components were detected by HPLC, it was explored how the material related to the pharmacological effects. In addition to offering alternate methods for KYD avoidance and management, this study will contribute to a deeper understanding of the possible mechanism variations of CD to avoid KYD.

## 2 Materials and methods

### 2.1 Reagents

Evo M-MLV reverse transcription, SYBR^®^ Green Pro Taq HS premix qPCR kit from Accurate Biotechnology(Hunan, China) Co.,Ltd. Propylthiouracil from Beijing Solarbo Technology Co., Ltd (Beijing, China). Triiodothyronine (T_3_), thyroid stimulating hormone (TSH), Tetraiodothyronine (T_4_), cyclic adenosine monophosphate (cAMP), thyrotropin releasing hormone (TRH), free fatty acid (FFA), lipoprotein lipase (LPL), Na^+^-K^+^-ATPase, nitric oxide (NO), cyclic guanosine monophosphate (cGMP), Ca^2+^-ATPase, hepatic glycogen (Glycogen), succinate dehydrogenase (SDH) kits were obtained from Shanghai Kexing Biotechnology (Shanghai, China) Co., Ltd. GAPDH, TRH, THRB1, THRA 1 + 2 antibody and HRP-IgG (H + L) from Beijing Bioss Biotechnology (Beijing, China) Co., Ltd. PBS from Dailian Meilun (Dailian, China) Co., Ltd. Acetonitrile and methanol of HPLC quality were acquired from Merck (China). Aladdin (China) supplied formic acid of HPLC quality. The Milli-Q system (Millipore, Bedford, MA, USA) was used to prepare ultrapure water. The remaining substances were all analytical grade.

### 2.2 Source of plants

*Cistanche deserticola* Y.C.Ma were collected from Alashan (Inner Mongolia, China) and authenticated by Professor Yanjun Zhai (Liaoning University of Traditional Chinese Medicine). Specimens of botanical drugs (CPU20190508-1, CPU20190508-2) were deposited in Liaoning Traditional Chinese Medicine Processing Technology Industry Innovation Center. Refer to the basis of our previous research (Liu et al., 2020), the optimal wine-steaming process parameters were used to prepare wine-processed CD.

### 2.3 Preparation and composition determination of CDG

500 g of CD/wine-processed CD was extracted with water three times, each for 1 h. The filtrates were combined, concentrated, and 95% ethanol was added to the appropriate concentration. After stirring, the solution was kept at 4°C overnight, then centrifuged. The supernatant was collected and concentrated until no ethanol remained. The solution was passed through a D101 macroporous resin column, and the eluate with 40% ethanol was collected and concentrated to obtain the total glycosides fraction. The total glycosides of raw CD [yield 7.96% (w/w)] and the total glycosides of wine-processed CD [yield 4.83% (w/w)] were stored in a refrigerator at 4°C. Utilizing HPLC-PDA(Waters, Milford, United States), chemical compound studies of the content of RG and WG extracts were carried out.

### 2.4 Animals and study design

Changsheng Biotechnology (Liaoning, China) Co., Ltd. was the commercial supplier of 40 male Sprague-Dawley (SD) rats weighing 200 ± 20 g and licensed under license number SCXK-(Liao) 2020-0001. Each rat was kept under SPF-standard lab conditions, which included full access to food and water, a temperature of 25°C ± 2°C, a humidity of 40% ± 10%, and a 12-hour cycle of light and dark. The rats were acclimated for 1 week before being randomly assigned to four groups (n = 8), which included the control group (CN), model group (MD), the 82 mg/kg/day total glycosides of raw CD treated group (RG), the 50 mg/kg/day total glycosides of wine-processed CD treated group (WG), and positive drug group (PS). The dosage was converted from the human dosage in the Chinese pharmacopoeia to the rat dosage according to the proportion of total glycosides of raw/wine-processed CD and the conversion of human and rat body surface area.

The animal studies were carried out in strict conformity with the standards of Chinese Experimental Animals Administration Legislation, and the Animal Care and Use Committee of Liaoning University of Traditional Chinese Medicine authorized the experimental protocol (NO. SYXK [Liao] 2019-0004). The rats were intragastrically administered propylthiouracil (10 mL/kg) for 15 days to induce the KYD rat model. The treatment group was given RG/WG extracts for 15 consecutive days, while the control (CN) and model group (MD) were given the same amount of saline. The positive group (PS) was given 0.936 g/kg Guifu Dihuang Pills.

### 2.5 Sample collections and measurements of visceral indexes and biochemical indexes

At the end of the 31st day, a minimum of 6 pills of feces were taken from every rat, separated into two sections in a sterilized EP tube containing 10 μL of 1% sodium azide (NaN_3_), and kept at −80°C for the 16S rRNA gene sequencing and feces metabonomics analyses that followed. After being put to sleep with a 20% urethane solution, the rats were killed. Rats from each group were slaughtered simultaneously. The plasma for the ELISA analysis was obtained by centrifuging certain blood specimens in a cylindrical centrifuge adding anticoagulant sodium for 15 min at 4°C, while other blood samples were kept at −80°C. The tissues of the liver, spleen, thymus, kidney and pathogenic axis (pituitary, thyroid, and hypothalamus) were quickly isolated on an ice surface, cleaned with saline, wiped with filter paper, frozen with liquid nitrogen, and stored at −80°. The thyroid was preserved for histological examination in 4% paraformaldehyde. Rats’ kidney, thymus, and spleen were measured in order to determine the organ index (Lian et al., 2024).

$$\text{Organ index}\left(\%\right)=\frac{\text{weight}\;\text{of}\;\text{the}\;\text{organ}\;\left(g\right)}{\text{weight}\;\text{of}\;\text{the}\;\text{body}\;\left(g\right)}\times 100\%$$

ELISA kits were used to measure the amounts of thyroid hormones, such as T_3_, TRH, TSH, FFA, T_4_, LPL, and NO in serum samples. A microplate reader was used to measure the amounts of Ca^2+^-ATPase, SDH, glycogen, and Na^+^-K^+^-ATPase of rat liver homogenate. Using ELISA kits, the amounts of key cell metabolic regulators, such as cAMP and cGMP in plasma samples were determined.

### 2.6 RT-qPCR analysis

Following the directions provided by the manufacturer, TRIzol reagent (Invitrogen, Carlsbad, CA, USA) was employed to extract total RNA of rapidly frozen hypothalamus and hypophysis tissues. Both the reverse transcription and genetic DNA removal reactions were carried out as directed. Amplification using SYBR^®^ Green Pro Taq HS qPCR was performed via an Applied Biosystems (ABI) 7500. Accurate Biotechnology (Hunan, China) Co., LTD generated the primer sequences for TRH and TSH using the Primer Premier 5.0 program, using the housekeeper gene β-actin as an internal reference. The following were the conditions for amplification: predenaturation at 95°C, denaturation at 95°C, and annealing at 60°C (30 s), with a total of 40 cycles. At the end of the reaction, the specificity of PCR amplification was determined by dissolution curve analysis, the Ct value was read, and the relative expression of target gene mRNA was calculated by the 2^−ΔΔCt^ method. Primer sequences are shown in Table 1.

**TABLE 1:** RT fluorescence quantitative PCR primers.

Genes	Primer sequence (5'→3′)	Length
*β-actin*	F:5′-GGAGATTACTGCCCTGGCTCCTA-3′	23
	R:5′-GACTCATCGTACTCCTGCTTGCTG-3′	24
*trh*	F:5′-TGTAAGCCCTGTATTCCCTATC-3′	22
	R:5′-AAGTCTCTGAAGTCGGTAAGG-3′	21
*tsh*	F:5′-CTACTGCCTGACCATCAACAC-3′	21
	R:5′-TCTGTAGGTAAAGTCTCTGTATGT-3′	24

### 2.7 H&E and immunohistochemistry staining

For the general histological test, 5 μm slices of the specimens were stained with hematoxylin and eosin (H&E) after they had been dried in an ordered sequence of alcohol and covered with paraffin. Lastly, a light microscope was used to view the thyroid gland’s abnormal appearance. The thyroid tissues were dehydrated and waxed, and then thin sections (4 μm) were blocked, deparaffinized, and placed for 12 h at 4°C with anti-Thyroid Stimulating Hormone Receptor (TSHR), anti-Thyroid peroxidase (TPO), and anti-Thyroglobulin (TG) (1:220; Abcam Technology, United Kingdom). As directed by the manufacturer, sections were incubated for 1 hour with secondary antibodies diluted 1:510 with horseradish peroxidase (HRP) labeled. To view immunological complexes, slides were cleaned and incubated for 10 min in PBS including 0.02% 3,3-diaminobenzidine and 0.01% H_2_O_2_. Finally, cells that had been positively labeled were examined under a light microscopy.

### 2.8 Metabolite purification along with profiling of fecal contents

After weighing 30 mg of the material into an EP tube, 500 μL of the resulting solution (methanol:acetonitrile:water = 2:2:1, a standard inner mixture that was isotopically labeled) was applied. These specimens were subsequently sterilized for 5 minutes in an ice water bath after undergoing homogenization for 4 minutes at 35 Hz. Three iterations of the homogenized and ultrasound cycle were conducted. Following a one-hour incubation period at −40°C, the specimens were spun up for 15 min at 4°C at 12,000 rpm. Quality control (QC) sample was prepared by mixing an equal aliquot of the supernatants from all of the samples. LC-MS/MS analyses were performed using an UPLC system (Vanquish, Thermo Fisher Scientific) with a UPLC BEH Amide column (2.1 mm × 100 mm, 1.7 μm) coupled to Q Exactive Orbitrap MS spectrometer (Thermo). Acetonitrile (A) and fluid at a pH of 9.75 containing 25 mmol/L ammonia hydroxide and ammonium acetate (B) made up the mobile phase. The auto-sampler temperature was 4°C. Since the QE HFX mass spectrometer can get MS/MS spectra in information dependent acquisition (IDA) mode under the supervision of the gathering system (Xcalibur, Thermo), it was utilized and the injection volume was 2 μL. During this mode, the data collection program constantly assesses the entire scan MS spectrum. The ESI source situations were established as follows: 3.7 kV positive and −3.0 kV negative spray voltage, 31 Arb sheath gas flow, 8000 MS/MS resolution, 24 Arb auxiliary gas flow, 350 °Ccapillary temperature, 65000 full MS resolution.

ProteoWizard was utilized to transform the raw data to the mzXML style, and an internal program built with R centered on XCMS was utilized for cleaning the data for maximum identification, mining, placement, and merging. Then, for metabolites annotation, an internal MS2 library (Biotree DB) was selected. Markup limit was determined at 0.3. For multidimensional statistical examination, all standard deviations were entered as unmodified information into the SIMCA 16.0.0 application (Sartorius Stedim Data Analytics, Umea, Sweden).

### 2.9 16S rRNA gene sequencing analysis

All rats’ feces were taken on days 0, 15, and 22 of the medication therapy. Following gathering, each feces specimen was quantified at 0.2 g (the mean across 5 specimens of feces), frozen right away using liquid nitrogen, followed by frozen at −80°C for further DNA extraction. As stated earlier, ERIC-PCR procedures, division, and whole genome DNA collection and evaluation have been carried out (Chi et al., 2019). The Illumina MiSeq platform was used to treat cleaned DNA specimens in preparation for 16S amplicon reading (Illumina, San Diego). Using barcoded 515F along with 806R broad primers designed for the V3-V4 zone as previously indicated, the 16S rRNA gene was selected for deletion (Abellan-Schneyder et al., 2021). In short, a restricted PCR cycle was utilized to combine the reads utilizing dual-index tags and Illumina DNA sequencing connections. The Nextera XT method was adopted for library normalization after purification with Agencourt AMPure beads (Agencourt, USA) (Rinke, 2018). Afterward, samples were placed into a single flow cell for sequencing on the MiSeq sequencing platform (Illumina, San Diego).

In the initial run, clumps were auto-generated using double tag reads having an average size of 2 × 300 bp and paired-end processed. PANDAseq was used to gather paired-end indicates and Raw FASTQ data were acquired in accordance with the preceding procedure (Langille et al., 2013). Barcodes and primers were removed from the sequences. “N” and any data sequences shorter than 250 bp were eliminated (Martijn et al., 2019). Solo readings and replicas were then eliminated after the processed scans grouped at k = 10 (97% similarity) (Zhang M. et al., 2023). Finally, BLASTn was used to OTUs with a selected library that was sourced via NCBI, RDPII, and GreenGenes (Li et al., 2022).

In short, isolate DNA using CTAB as directed by the manufacturer. PCR was utilized to amplify the 16S rRNA gene’s V3-V4 domain. An Agilent 2100 bioanalyzer (Agilent, USA) and an Illumina Novaseq platform were used for the sequencing process. Software called VSearch (v2.3.4) can be used to filter hybrid genomes. Following DADA_2_ removal, we were left with a characteristic list and a metabolite series. The Silva (version 138) classifier states that the characteristic levels are normalized using each specimen’s relative abundance. After dereplication using DADA_2_, we obtained feature table and feature sequence. According to SILVA (release 138) classifier, feature abundance was normalized using relative abundance of each sample. Five indices—Chao1, observed species, goods coverage, Shannon, and Simpson—are used to analyze the degree of species diversity. QIIME2 was used to calculate each index for our collections. QIIME2 was utilized for assessing beta diversity, and the R program was used to create the visualizations. Aligned sequence alignment was done using Blast, and every instance sequence’s characteristic regions were added to using the SILVA library. The R package (version 3.5.2) was used to create additional graphics. (Xie et al., 2024).

### 2.10 Analyzing metabolic pathways and identifying possible biomarker

KEGG, HMDB, METLIN and MetaboAnalyst were used to import the different metabolites that were picked from feces metabonomics for pathway enriched research. Through the application of the PICRUSt 2 program to compare the variety makeup parameters derived through the 16S sequenced data, the practical structure of microorganism specimens was calculated, allowing for the analysis of the practical distinctions between various specimens or groups (Langille et al., 2013). Lastly, applying the G-TEST and Fisher test procedures in the STAMP apps, a major distinction test among specimens was conducted at the degree of genus for biodiversity between various specimens (t-test, *P* < 0.05). Generally speaking, comparative examinations of KEGG metabolic pathways can predict variations and shifts in the biochemical pathways associated with function genes of microbial communities across samples within other groups.

### 2.11 Characterization of the association between gut microbiotas and metabolites

A Pearson correlational study was conducted between the genus of disturbed gut microorganisms assessed from 16S rRNA gene sequencing analysis and the modified metabolites filtered from fecal metabonomics (Zhao et al., 2022). Those correlation coefficient values (|r| ≥ 0.70, *P* < 0.05) were considered to be significantly correlated. Eventually, significant related metabolites and gut microbe genus were obtained and displayed as heat maps (*P < 0.05, **P < 0.01). Red indicates a positive correlation while blue indicates a negative correlation.

### 2.12 Statistical analysis

The findings were displayed as mean ± SEM, with the number of repetitions for each group indicated in the caption. Software named SPSS 20.0 (IBM, USA) was used to conduct the statistical analysis. The degrees of statistical validity were ascertained using the LSD test and one-way ANOVA. The threshold for the level of significance was chosen *P* < 0.05 or *P* < 0.01. For intestinal bacterial study analysis, GraphPad Prism (GraphPad Prism version 5.1) was utilized for statistical examination and graphical representation of the data. To identify substantial variations, a Kruskal-Wallis test with Bonferroni correction was employed. For alpha and beta diversity analysis, the R packages phyloseq, ggplot_2_ and scales were utilized. For multidimensional statistical evaluation, including PCA, PLS-DA and OPLS-DA, all normalized parameters were entered as raw data into the SIMCA-P 13.0 software (Umetrics, Sweden). The PLS-DA model has been confirmed using the method of 200 permutation testing, OPLS-DA was validated using both an a whopping seven a cross-valid and an ANOVA of the CV-ANOVA technique.

## 3 Results

### 3.1 HPLC analysis of RG and WG

**FIGURE 1:**
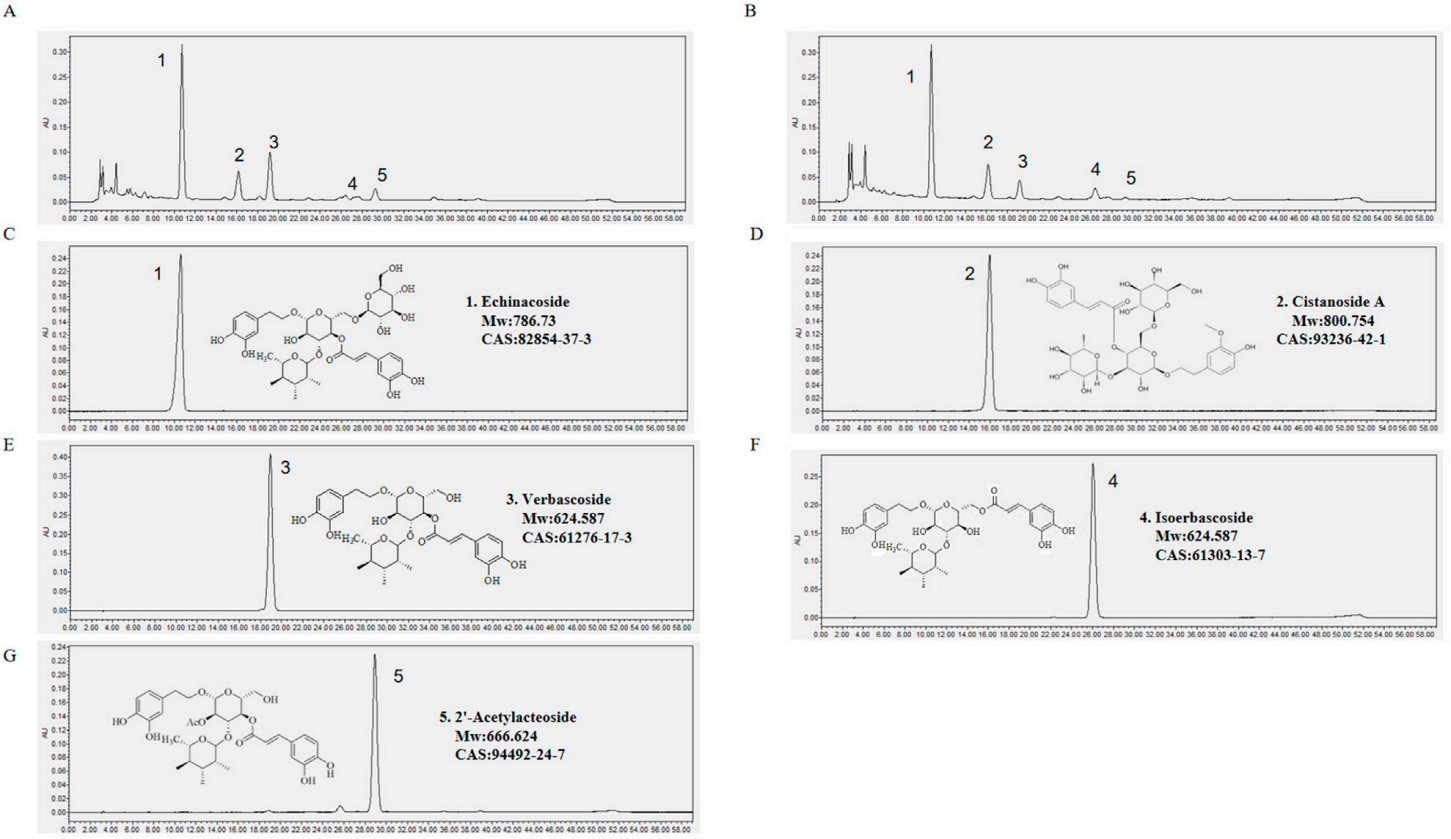
RG and WG symbolic constituent chromatograms. **(A)** RG chromatogram. **(B)** WG chromatogram. **(C)** Chromatogram of Echinacoside. **(D)** Chromatogram of Cistanoside A. **(E)** Chromatogram of Verbascoside. **(F)** Chromatogram of Isoverbascoside. **(G)** Chromatogram of 2′-Acetylacteoside. Observation wavelength (λ) = 330 nm.

Both RG and WG have phenylethanoid glycoside compositions that differ significantly, the contents of echinacoside, acetoside, and 2′-acetylacteoside decrease, while the content of isoacetoside increases significantly (Figure 1). Our prior research commented on the transition process (Li et al., 2021).

### 3.2 Effects of CDG on HPT axis pharmacodynamic analysis in rats with KYDS

#### 3.2.1 Conventional indicators

**FIGURE 2:**
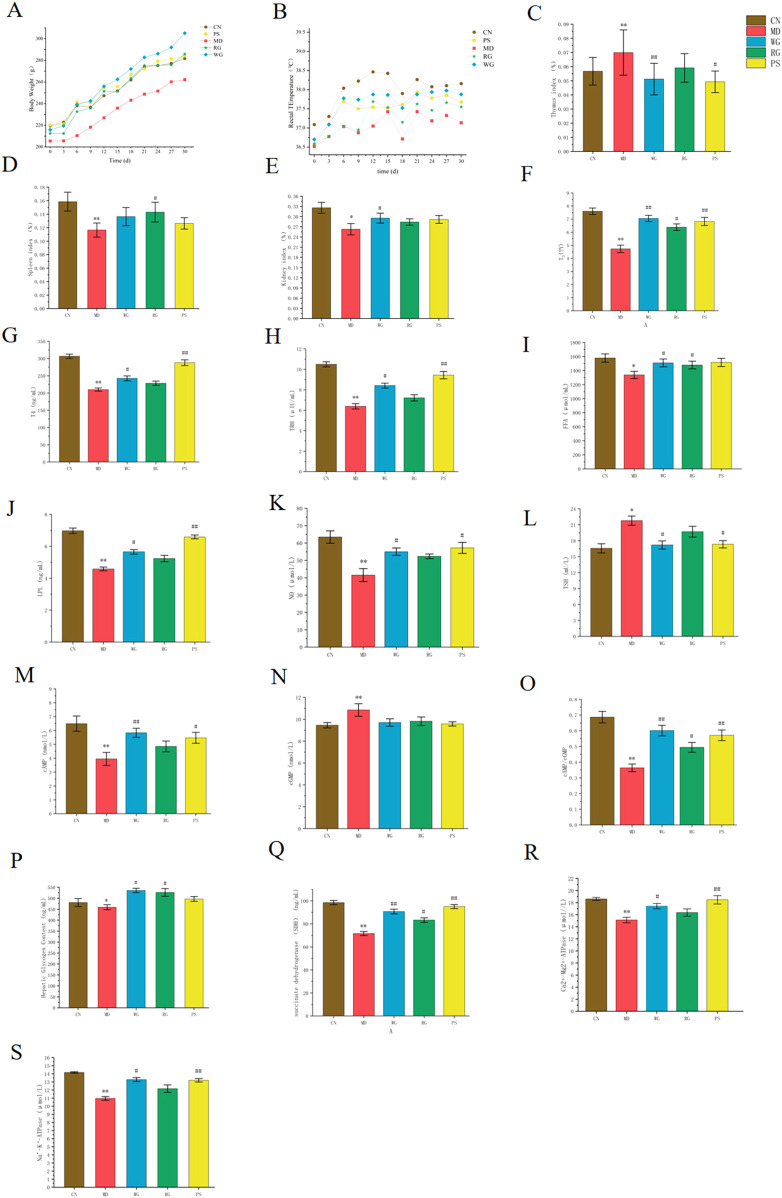
The impact that CDG has on body temperature, body weight, organ index, serum, plasma, liver homogenate related to energy metabolism index cytokine. **(A)** Random body weight of rats at 30 days. **(B)** Random body temperature of rats at 30 days. **(C)** Thymus indexes. **(D)** Spleen indexes. **(E)** Kidney indexes. **(F)** Serum T_3_ levels. **(G)** Serum T_4_ levels. **(H)** Serum TRH levels. **(I)** Serum FFA levels. **(J)** Serum LPL levels. **(K)** Serum NO levels. **(L)** Serum TSH levels. **(M)** Plasma cAMP level. **(N)** Plasma cGMP level. **(O)** Plasma cAMP/cGMP level. **(P)** Concentration of energy metabolism index cytokine glycogen. **(Q)** Concentration of SDH. **(R)** Concentration of the Ca^2+^-ATPase. **(S)** Concentration of Na^+^-K^+^-ATPase.MEAN values ± SD (n = 6) were utilized to display the data. The student’s test was employed for statistical analysis. *P < 0.05, **P < 0.01, ***P < 0.005 in comparison to the CN group. In contrast to the MD group, ^#^ *P* < 0.05, ^##^ *P* < 0.01, ^###^ *P* < 0.005.

Rats in the MD group exhibited characteristics such as fatigue, anorexia, aversion to cold, polyuria, reduced activity, and delayed response after being administered with propylthiouracil. Compared to the CN group, the MD group’s body weight increased at a substantially slower rate (Chen et al., 2019). After 20 days, the rats in the PS group’s weights steadily increased while the animals from the other drug groups’ body weight kept rising. Rats in the MD group had a lower rectal temperature than those in the CN group, however following injection, the rats’ stools rose. Rats’ spleen and kidney indices both sharply declined following propylthiouracil treatment, whereas their thymus indices increased. Following WG treatment, the thymus index dropped compared to RG treatment, while the spleen and kidney indices of KYD rats grew greatly (Figures 2A–E).

#### 3.2.2 Indices based on biochemistry

TSH content rose whereas T_3_, T_4_, TRH, FFA, LPL, and NO levels dramatically dropped in the MD group as opposed to the CN group. In the MD group, the quantity of cGMP rose while the plasma amounts of cAMP and cAMP/cGMP sharply declined. Level of glycogen, SDH, Ca^2+^-ATPase, Na^+^-K^+^-ATPase in liver homogenates in the MD group reduced. After the intervention of RG, the content of T_3,_ T_4_, TRH, FFA, LPL, NO cAMP, cAMP/cGMP, glycogen, SDH, Ca^2+^-ATPase and Na^+^-K^+^-ATPase in the KYD rats increased, while the concentration of TSH, cGMP decreased. Compared to the RG group, the WG group’s concentrations of T_3,_ T_4_, TRH, FFA, LPL, NO cAMP, cAMP/cGMP, glycogen, SDH, Ca^2+^-ATPase and Na^+^-K^+^-ATPase were clearly elevated, although TSH and cGMP were the opposite.

Among these, cAMP, cGMP were specific indicators for diagnosis of KYD. T_3_, T_4_, TRH and TSH were indicators that reflected thyroid function in HPT axis. While SDH, Ca^2+^-ATPase and Na^+^-K^+^-ATPase were functional indicators of energy metabolism. FFA and LPL were functional indicators that reflected fat metabolism (Cheng et al., 2024). Glycogen was an important indicator of glucose metabolism. NO is an indicator of kidney oxidative damage (Cao et al., 2024). These results showed that RG,WG may alleviate thyroid function on the HPT axis by modulating TRH, T_4_, TSH and T_3_, alleviate oxidative damage in kidney tissue by modulating NO, and improve energy metabolic function in rats with KYD by modulating SDH, Ca^2+^-ATPase, Na^+^-K^+^-ATPase, glycogen, FFA and LPL (Figures 2F–S) (Zhu et al., 2022).

#### 3.2.3 Histopathological analysis and RT-qPCR

**FIGURE 3:**
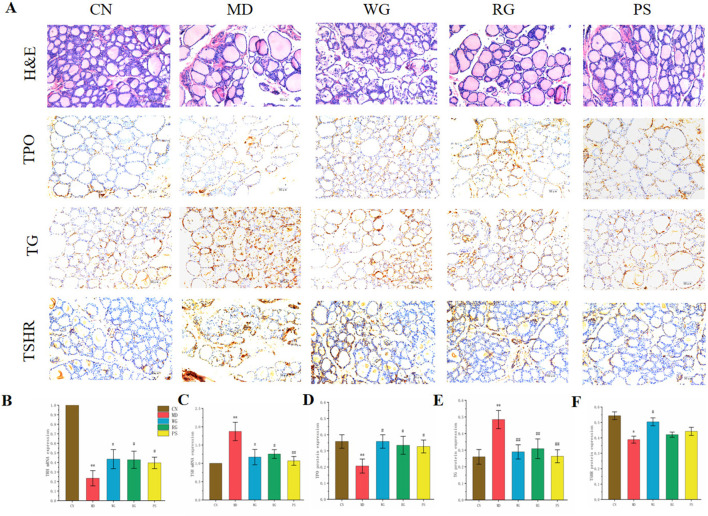
Effect of CDG on **(A)** H&E staining and immunohistochemical expression of **(D)** TPO, **(E)** TG and **(F)** TSHR proteins in the thyroid of KYD model rats. Scale bar = 50 μm, original magnification 50×. **(B)** Expression of TRH mRNA in the hypothalamus **(C)** TSH mRNA expression in pituitary gland of rats in each group.

As shown in Figure 3A, in the KYD group (MD): The number of thyroid cells decreased, the intercellular space widened, the colloid in the thyroid follicular cavity decreased, the cell shape was irregular, and vacuolar degeneration occurred. While TG protein expression greatly rose (*P* < 0.01), TPO and TSHR protein expression substantially reduced (*P* < 0.05). Following CDG administration, there was an upward trend in TPO and TSHR expression. There was a downward trend in TG expression. The quantification of TPO, TSHR, and TG in the PS group and WG group differed markedly from that of the MD group (Figures 3D–F). Rats in the MD group had considerably lower TRH mRNA expressions in their hypothalamus than rats in the CN group (*P* < 0.01). Following treatment, the RG group’s hypothalamic TRH mRNA expressions were substantially greater than those of the MD group (*P* < 0.05), while the WG group’s were more noticeable (*P* < 0.01) (Figures 3B,C). The expression of TSH in pituitary of KYD rats was opposite. The expression of TSH was significantly decreased after CDG treatment, and WG was more obvious than RG.

### 3.3 Investigation of metabolism

#### 3.3.1 CDG reversed the metabolic profile of KYD rats

**FIGURE 4:**
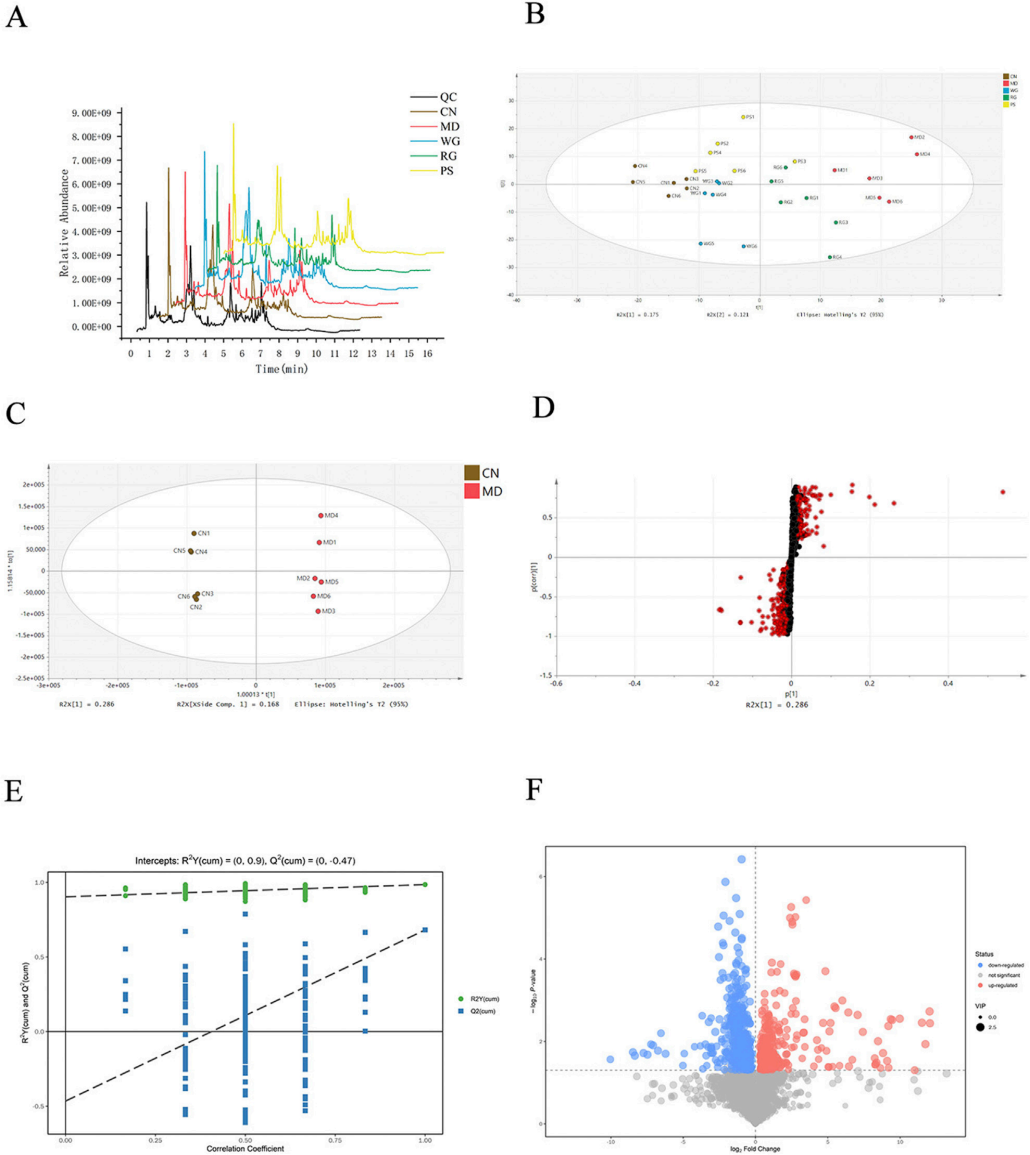
Analysis of fecal samples’ metabolism. **(A)** In the positively charged ionic type of stool samples, a typical TIC of the QC, CN, MD, WG, RG, and PS groups. **(B)** Plots of the five groups’ PLS-DA scores according to their fecal metabolism characteristics. **(C)** Plots of the CN and MD groups’ OPLS-DA scores. **(D)** The OPLS-DA model’s S-plot of the CN and MD group. As indicated by the red dot, VIP >1.0. **(E)** Fecal OPLS-DA permutation test. **(F)** Plot of the Feces Volcano. Variable metabolite upregulation is indicated by red. Variable metabolic downregulation is indicated by blue. Metabolites with no statistical difference are shown in gray.

To comprehensively clarify the mechanism of Cistanche’s total glycosides on improving KYD, all specimens of stool were analyzed in positive-ions mode using UPLC-MS/MS. Under perfect circumstances, the TIC of feces samples from 5 groups were acquired (Figure 4A).

Multivariate analysis revealed significant differences among all five groups. PCA model and the profiles of the CN, MD, PS, RG and WG group showed a trend of separation (Supplementary Figure S1). Further PLS-DA analysis was performed to distinguish the five groups of metabolites, and the PLS-DA score plot showed that these groups can be easily distinguished (Figure 4B). This suggests that the naturally occurring metabolites in feces vary significantly throughout groups. Propylthiouracil-induced KYD rats were successfully established, as evidenced by the separation of the CN and MD. The WG and RG groups’ symbols were completely distinct from the MD group, suggesting every treatment group had various degrees of control over the propylthiouracil-induced metabolic issue in KYD rats and that the current pattern of regulating was WG > RG group.

#### 3.3.2 Identification of potential endogenous biomarkers

To explore potential biomarkers related to KYD. As shown in Figure 4C, in the OPLS-DA score plot, there was a good separation between the CN group and the MD group, revealing the obvious changes of metabolites in the KYD rats induced by propylthiouracil, and indicating that the KYD model had completely different metabolic characteristics compared with the CN group. To assess the explanation rate (R^2^Y), forecasting capacity (Q^2^), and other model characteristics, a random permutation test (n = 200) was conducted under the well-established OPLS-DA model. R^2^ = 0.9, Q^2^ = −0.470 (Figure 4E). The findings indicate that there is no excessive fitting phenomena and that the model has excellent stability and forecasting capability (Shan et al., 2021). KYDS biomarkers have been assessed using S-plots. The KEGG, mzCloud, ChemSpider and HMDB database were applied to identify internal metabolites (Yu et al., 2024). By connecting the S-plots plot with the VIP>1 and T-test (*P <* 0.05) in the volcano plot (Figures 4D,F), the largest impact characteristics’ divergent factors were identified as KYD syndrome-related biomarkers. Following the identification of the MS-MS outcomes in the web-based database, ion features with VIP>1.0 and T-test (*P <* 0.05) were evaluated as correlates linked to KYDS. Supplementary Table S1 displays the findings.

**FIGURE 5:**
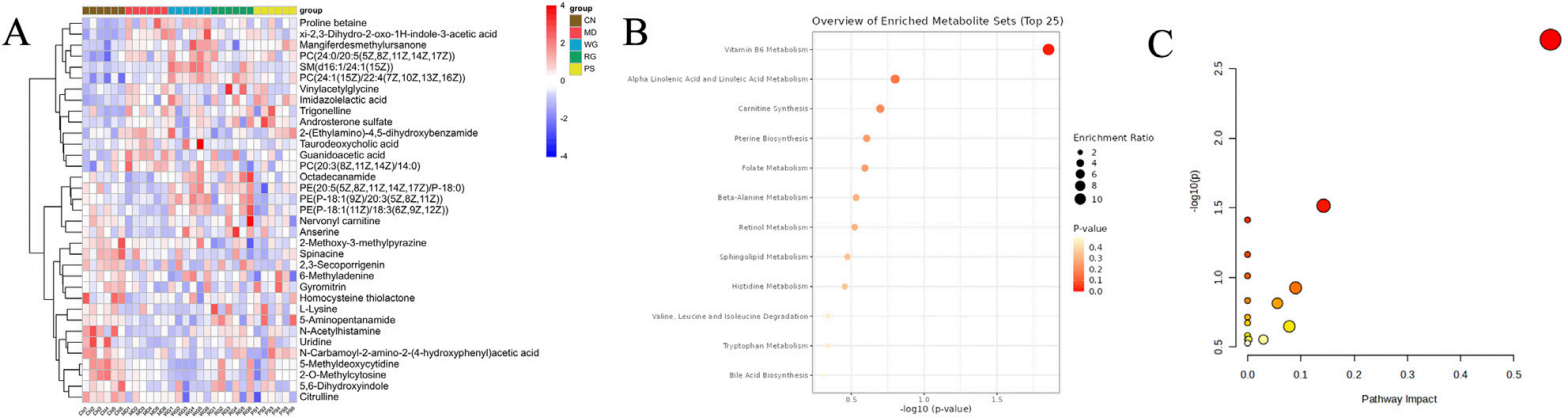
Path evaluation of KYD metabolites that have been considerably altered. **(A)** The degree of change is indicated in upregulation and downregulation on the hierarchical grouping heat map of the fecal differing metabolites. **(B)** Visual examination of the concentration route of modified chemicals in feces. **(C)** Examination of the fecal route of common metabolites in exposure to KYD. A metabolic route is represented by each circle.

A combined number of 98 metabolites were seen modified in the MD group as in contrast to CN in fecal metabolites (Figure 5A). The 40 metabolites’ concentrations including L-cis-3-Amino-2-pyrrolidinecarboxylic acid,4,6-Dihydroxyquinoline,N-Acetyl-D-glucosaminyldiphosphodolichol, (+)-2,3-Dihydro-3-methyl-1H-pyrrole,Isonicotinic acid, 3-Hydroxymethylantipyrine, Laccarin, Glycerophosphocholine,Aldosine, N′-Hydroxymethylnorcotinine,Spirostane-3,6-dione, Octadecanamide, Erinacine D,N-a-Acetylcitrulline and Simvastatin were decreases in the MD. But compared to the MD group, the RG and WG groups had higher levels of over 17 and 24 metabolites, correspondingly. Remarkably, the MD rats’ levels of 58 metabolites were higher, including 6-Methylnicotinamide, Nornicotine, 6-Hydroxy-1H-indole-3-acetamide,Thyrotropin releasing hormone, Isopropylpyrazine, 4-Trimethylammoniobutanal, Pyridoxal, Pyridoxamine, Genipinic acid, 5′-Hydroxycotinine,Cytokinin B,Lysyl-Lysine,sn-Glycero-3-phosphocholine,P hosphatidylcholine, 3-(3,4-Dihydroxyphenyl)-2-methoxypropionic a,Benzenepropanenitrile, Carnosine, Folate, 4-Trimethylammoniobutanal, Sphinganine, Isonicotinic acid, 4,6-Dihydroxyquinoline, Calcitriol, N-Acetyl-D-glucosaminyldiphosphodolichol,beta-Vatirenene, Ercalcitriol, Smilagenone, Folic acid, Endomorphin-2 and Androsterone sulfate compared with the CN group, while the RG and WG group had lower levels of the 42 and 46 metabolites than the model group, respectively. In addition, RG and WG also showed some differences in the regulation of metabolites related to KYD. Taurodeoxycholic acid, proline-betaine, guanidoacetic acid, 5-(Ethylamino)-4,5-dihydroxybenzamide, 5,6-Dihydroxyindole, L-lysine, and 5-Aminopentanamide were all more effectively modulated by RG. WG mostly controls vinylacetylglycine, guanidoacetic acid, 2-(Ethylamino)-4,5-dihydroxybenzamide, 6-methyladiene, and anserine (Supplementary Figure S2). Supplementary Figure S3 displayed the association analysis between those metabolites. In conclusion, RG as well as WG dramatically reversed the aberrant expression of the subsequent metabolites linked to KYD as well as 70 frequent indicators: Folate, Glycerophosphocholines, Amino acids, Vitamin B6, Linoleic acid, Fatty amides, Indolyl carboxylic acids and derivatives, etc.

### 3.4 Analysis of metabolic pathway of potential biomarkers

**FIGURE 6:**
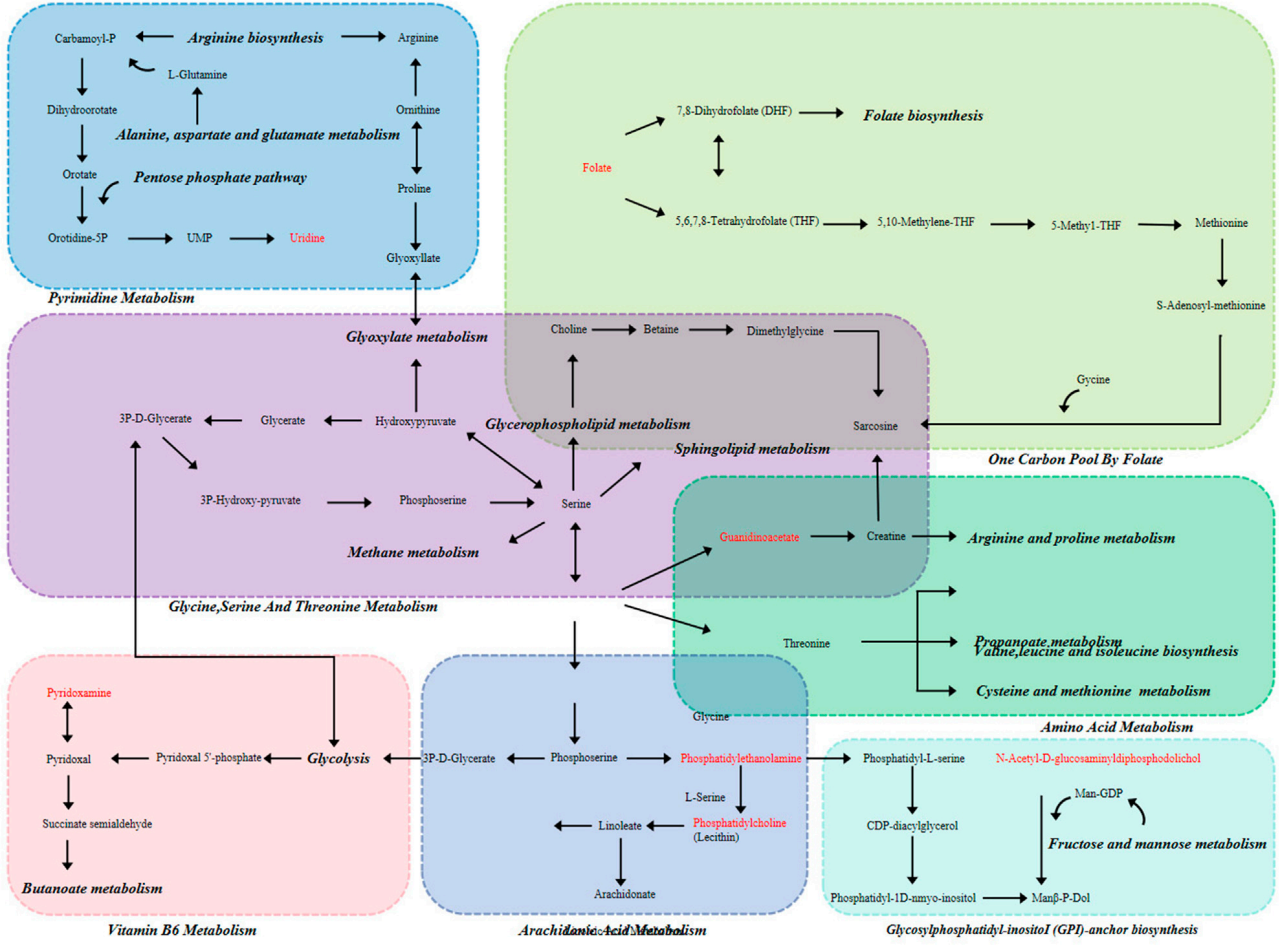
Network of potential biomarker changes based on KYD and CDG regulation. Arrows point upward or downward. WG was marked by blue, and RG by green.

For the purpose of metabolism evaluation, all of the aforementioned putative indicators were imported into MetaboAnalyst (Xu et al., 2023). These pertinent paths were found using parameters (effect value >0.1) as the ranking factor. As seen in Figures 5B,C, the outcome was displayed as an interactive visual system. Pyrimidine, one carbon pool by folate, amino acid, vitamin B6, arachidonic acid, glycine, serine, threonine, GPI-anchor biosynthesis metabolism were the metabolic pathways that were strongly linked to the pharmaceutical regimen of propylthiouracil-induced KYD rats, according to the outcomes of metabolic pathway enhancement. Figure 6 displayed the main routes of metabolism linked to biomarkers. We examined the reaction of metabolites disrupted by modeling formation following CDG treatment in order to assess the curative effect and explain the mechanisms of CDG on KYD rats. Among the possible biomarkers linked to KYD were Guanidoacetic acid, Uridine, Lecithin, Phosphatidylethanolamine, N-Acetyl-D-glucosaminyldiphosphodolichol, Pyridoxamine, Folic acid. The variation trends of typical metabolites were shown respectively in Supplementary Figure S4. Compared with the MD group, the levels of Guanidoacetic acid, Lecithin, N-Acetyl-D-glucosaminyldiphosphodolichol, Pyridoxamine and Folic acid were increased, after RG and WG treatment, which was close to CN group, especially for WG treatment.

### 3.5 Regulation of CDG on the intestinal flora diversity of KYD rats

**FIGURE 7:**
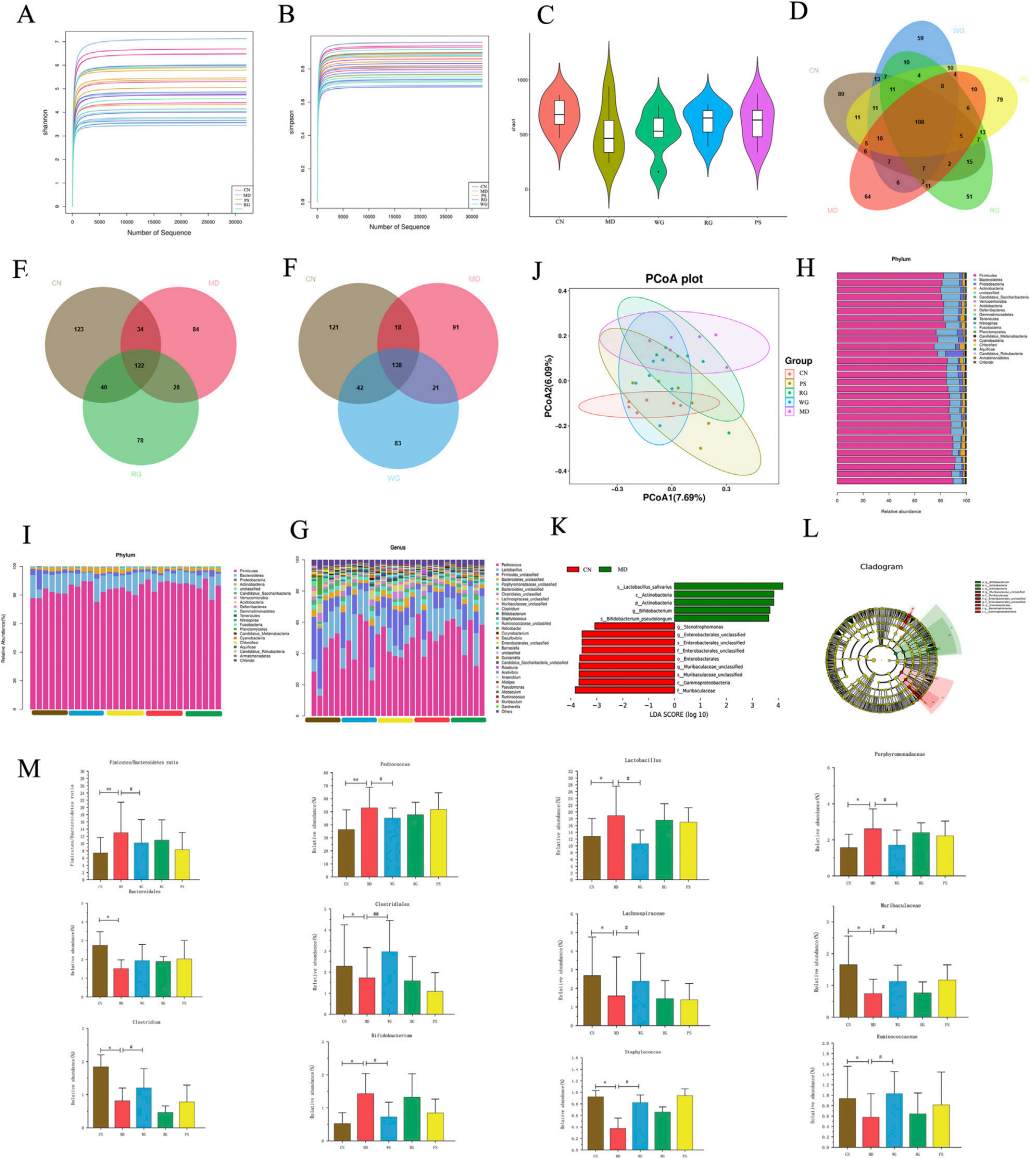
The variations in each group’s gut microbes. **(A)** The Shannon index used to determine the diversity of bacteria. **(B)** The Simpson index utilized for assessing variation in bacteria. **(C)** Bacterial richness estimated based on the Chao 1 value **(D–F)** Veen diagram of ASV clustering: The value inside the ASV in this area represents the number of ASVs. CN, MD, WG, RG and PS groups were marked brown, red, blue, green, yellow, respectively. **(J)** PCoA analysis. **(H)** The phylum level mean proportions of bacterial species. **(I)** Phylum level characterization of the microbiota in the gut by bacterium species. **(G)** Genus level characterization of the intestinal microbes by bacterium species. **(K)** Biodiversity cladogram produced by LEfSe study. **(L)** Taxonomic scores from LDA (log10) (score>3), the changes of differential bacteria in each group (n = 6). **(M)** Ratio of *Firmicutes*-to-*Bacteroidetes* and abundance of the key differentiated gut microbiota at genus level. Compared with CN group, *P < 0.05, **P < 0.01. Compared with MD group, ^#^ *P* < 0.05, ^##^ *P* < 0.01.

Figure 7 displays findings of the 16S rRNA technology assessment of the gut bacteria. The Shannon and Simpson curves remained relatively flat as the sequencing amount increased. This trend suggests that the sequencing data are robust and reliable. The species within the sample are not only abundant but also hardly distributed, strongely capturing underlying units structures (Figures 7A,B). Alpha diversity primarily reflects species richness and evenness through the Chao1 index (Figure 7C). In this study, Thirty samples were sequenced using Illumina MiSeq sequencing to produce original gene tags, and the paired-end information was spliced using the overlaid approach for chimeric sequence identification and quality control. In the Venn diagram of rat intestinal microbial diversity (Figure 7D), 108 shared ASVs were identified across the five groups. The overlap between the WG and CN groups was larger than that between the RG and CN groups, indicating that the WG group is more similar to the CN group. These findings suggest that CDG has the potential to ameliorate the disorder of the microbiota structure in KYD rats, with the WG treatment demonstrating a more pronounced effect (Figures 7E,F). Principal Coordinate Analysis (PCoA) results are presented in Figure 7J. The first two principal coordinates (PCoA1 and PCoA2) account for 7.69% and 6.09% of the total variance, respectively.

Figures 7H,I displayed the composition of the phylumlevel flora in every group. *Firmicutes* and *Bacteroidetes* made up almost all of the gut bacteria among all groups. While the total amount of *Bacteroides* in all groups receiving treatment was substantially less than that of the MD group (*P* < 0.05), the richness of *Bacteroides* in the MD group was 87.2%, which was far greater than that of CN group (80.1%). The percentage of *Firmicutes* to *Bacteroidetes*, which usually reflects the composition of the main intestinal flora (Li J. et al., 2024), was 7.52,13.22,10.51,11.31 and 8.31 in each group, respectively (Figure 7M). Based on LEfSe analysis, it was found that the abundance of *Actinobacteria* and *Chloroflexi* in MD group was significantly increased compared with CN group, while the abundance of *Actinobacteria* and *Chloroflexi* in other treatment groups was decreased compared with MD group. In addition, propylthiouracil intervention resulted in a significant reduction in the abundance of *Bacteroidetes* and *Fusobacteria* in the intestine of rats, which could be reversed by CDG intervention (Figures 7K,L). In conclusion, CDG improves the disturbance and structural changes of kidney-yang deficiency microflora induced by propylthiouracil at the phylum level.

The top 10 species in each group were identified by additional bacteria analysis at the general level in terms of sample organization and range (Figure 7G), comprising, *Pediococcus*, *Lactobacillus*, *Firmicutes*, *Bacteroidetes*, *Porphyromonadaceae*, *Bacteroidales*, *Clostridiales*, *Lachnospiraceae*, *Muribaculaceae*, *Clostridium* and *Bifidobacterium*, which were dominant among all groupings. The predominant bacteria found in the CN group were *Firmicutes, Lactobacillus*, *Pediococcus*. Crucially, the MD group had a considerably larger amount of *Lactobacillus* than the rest of the groups (*P* < 0.05). Figure 7M depicts that in MD, the number of *Bifidobacterium*, *Lactobacillus*, *Porphyromonadaceae* and *Pediococcus* increased significantly, while the number of *Bacteroidales*, *Clostridiales*, *Lachnospiraceae*, *Muribaculaceae*, *Clostridium*, *Staphylococcus* and *Ruminococcaceae* decreased significantly. On the contrary, in the RG/WG treatment group, the abundance trends of 11 genera such as *Bifidobacterium, Bacteroidales*, *Clostridiales*, *Lachnospiraceae*, *Muribaculaceae* were reversed (*P* < 0.05). The relative abundance of the microbial community had a greater impact by the WG group than by the RG group.

### 3.6 Correlation analysis between gut microbiota, fecal metabolic and HPT axis ELISA indicators

**FIGURE 8:**
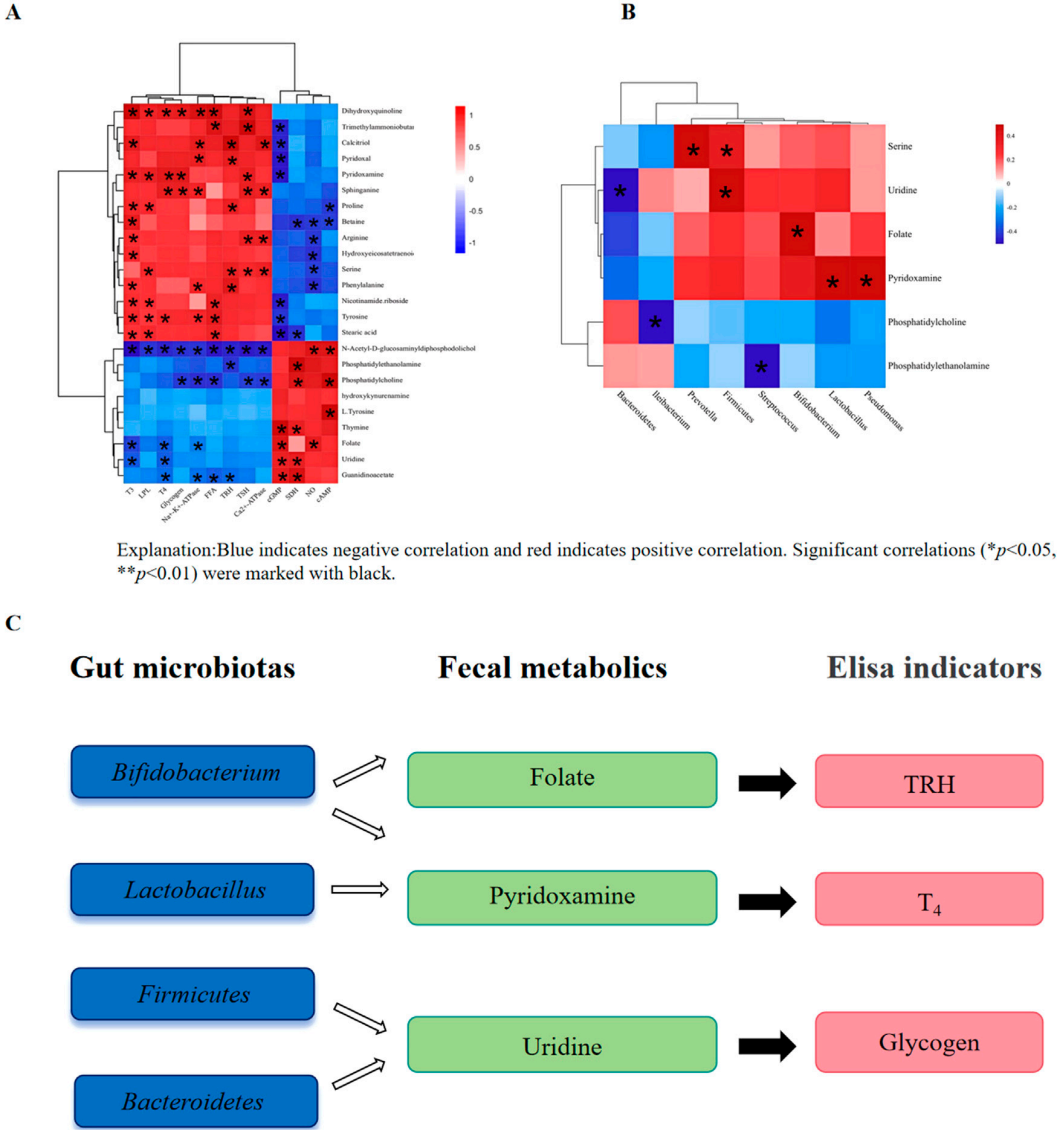
The following associations were found: **(A)** various metabolites and HPT axis ELISA indicators (the red/blue colors showed a positive/negative correlation); **(B)** differential metabolites and intestinal flora; **(C)** possible fecal metabolites mediating the possible relationship between intestinal flora and HPT axis ELISA indicators.

Fecal metabolites mediate the interaction between gut microbiota and HPT axis ELISA indicators in rats. Metabolites play an important role in host-microbiome interactions. First, we used pearson correlation analysis to evaluate HPT axis ELISA indicators and key fecal metabolites. As shown in Figures 8A, T_3_, T_4_ and TRH were positively correlated with Pyridoxamine, Tyrosine, Stearic acid and Serine, while Glycogen was strongly negatively correlated with Uridine, Folate and Phosphatidylcholine. These data suggested that RG/WG may play a role in anti-KYD through regulating specific fecal metabolites. Secondly, in order to better understand the functional association between fecal metabolites that interact with intestinal flora, correlation analysis was performed on bacterial genera and metabolites with significant differences. As shown in Figure 8B, Serine was positively correlated with *Prevotella*, Folate was positively correlated with *Bifidobacterium*, Pyridoxamine was positively correlated with *Lactobacillus*, similar to Uridine and *Firmicutes-*to*-Bacteroidetes*, Pyridoxamine and *Pseudomonas*. RG/WG may significantly improve KYD rats through the connection between intestinal flora and arachidonic acid metabolism. For example, the observed Phosphatidylcholine between intestinal flora and fecal metabolites was negatively correlated with *lleibacterium*, and Phosphatidylethanolamine was significantly negatively correlated with *Streptococcus*.

## 4 Discussion

Traditional Chinese Medicine (TCM) posits that KYD represents a fundamental imbalance characterized by insufficient yang energy, clinically manifested through a constellation of symptoms including aversion to cold, cold extremities, fatigue, lumbosacral soreness, sexual dysfunction, and polyuria with clear urine (Jin et al., 2025). Modern pathophysiological investigations have established significant correlations between KYD patterns and the development of chronic disorders such as chronic nephritis, adrenal insufficiency, chronic prostatitis, and infertility (T_3_/T_4_). Central to this dysregulation lies hypothalamic-pituitary-thyroid (HPT) axis dysfunction, as evidenced by animal model studies demonstrating reduced thyroid-stimulating hormone (TSH) and thyroid hormone levels that parallel the metabolic manifestations of KYD. The observed hypothyroid state induces decreased basal metabolic rate, directly contributing to characteristic thermoregulatory dysfunction and lethargy (Ayu et al., 2020). Furthermore, disrupted TSH feedback inhibition suggests impaired hypothalamic regulation of thyroid homeostasis, exacerbating the metabolic disequilibrium in KYD pathogenesis. Despite these mechanistic insights, the precise neuroendocrine-immune interactions underlying KYD remain incompletely defined (Li et al., 2020).

Our current investigation systematically addresses this knowledge gap through induced KYD animal models. Our findings reveal significant splenomegaly, as indicated by elevated splenic indices, suggesting chronic inflammatory activation-a novel observation requiring further immunological characterization. Conversely, thymic and renal atrophy, demonstrated by reduced organ indices, implies structural degeneration and functional decline in immune regulation and endocrine homeostasis (Wachsmuth et al., 2022). These morphometric alterations provide valuable morphological correlates for the previously described metabolic disturbances, establishing a foundation for exploring the bidirectional relationships between HPT axis dysregulation and organ remodeling in KYD progression.

The concurrent reductions in serum triiodothyronine (T_3_), thyroxine (T_4_), thyrotropin-releasing hormone (TRH), free fatty acids (FFA), lipoprotein lipase (LPL), and nitric oxide (NO) levels are consistent with the hallmark metabolic depression and energy deficit characteristic of Kidney Yang Deficiency (KYD). The observed suppression of the cAMP/cGMP ratio suggests impaired signaling pathways involving cAMP-dependent protein kinase A (PKA) and guanosine monophosphate-dependent protein kinase G (PKG), which are crucial regulators of energy metabolism and vascular homeostasis. The depletion of hepatic glycogen further corroborates the disruption of gluconeogenesis and glycogenolytic reserve, aligning with systemic energy failure. The coordinated inhibition of mitochondrial enzymes, including succinate dehydrogenase (SDH, Complex II), Na^+^-K^+^-ATPase (for membrane ion gradient maintenance), and Ca^2+^-ATPase (for intracellular calcium homeostasis), provides direct evidence of bioenergetic collapse at the cellular level. These biochemical disturbances collectively indicate that hypothyroidism driven by the hypothalamic-pituitary-thyroid (HPT) axis, through reduced T_3_/T_4_-mediated mitochondrial biogenesis, leads to decreased thermogenesis. The dysfunction of the HPT axis initiates a cascade of metabolic derangements that manifest as thermoregulatory failure and energy depletion in KYD (Ayu et al., 2020; Li et al., 2020).

The fecal metabolomics results of KYD rats treated with CDG showed that KYD was associated with 98 different metabolites and 8 possible pathways for metabolism. These biochemical processes and metabolites were mostly engaged in the metabolism of vitamin B6, glycerophospholipids, linoleic acid, one carbon pool by folate, lysine degradation, arachidonic acid, histidine, and ether lipids. The metabolism of vitamin B6, arachidonic acid, and folic acid were the key routes of metabolism. Fecal metabolomics showed that Pyridoxamine, L-Serine, Phosphatidylethanolamine, Phosphatidylcholine and Folate were significant biomarkers. Compared with the CN group, Pyridoxamine, Phosphatidylcholine and Folate increased; L-Serine. Phosphatidylethanolamine decreased in the MD group. Different degrees of reversal occurred after RG/WG treatment. Among them, WG downregulated Pyridoxamine, Phosphatidylcholine and Folate; and upregulated L-Serine and Phosphatidylethanolamine. Research has indicated that the primary network responsible for producing inflammatory mediators and inducing inflammation is the metabolic network of arachidonic acid, which is engaged in the body’s immunological response; secondly, vitamin B6 metabolism is involved in a variety of metabolic reactions in the body, which can regulate amino acid metabolism, immune system, neurotransmitter synthesis, etc., which is closely related to the clinical manifestations of KYD (Hui et al., 2023).

Through 16S rRNA gene sequencing analysis, we found that the intestinal microbiota after modeling was different at the level of phylums and 11 different genera. The main differential genera were *Prevotella*, *Lactobacillus*, *Bifidobacterium*, *Pseudomonas*, *Firmicutes*, *Bacteroidetes*. Previous studies have found that *Prevotella* is related to the peripheral thyroid homeostasis of the HPT axis, which increases in hyperthyroidism and decreases in hypothyroidism (Virili et al., 2021). *Lactobacillus* and *Bifidobacterium* play an important role in the pathogenesis and serological diagnosis of autoimmune thyroid diseases (Kiseleva et al., 2011). *Bifidobacterium* can synthesize a variety of digestive enzymes to assist the body’s digestion and absorption in the gastrointestinal tract (Huang et al., 2024). *Bifidobacterium* is directly or indirectly involved in the synthesis, absorption and utilization of vitamin B (Tindjau et al., 2024). *Pseudomonas* is an important indicator for the diagnosis of hypoimmunity caused by pituitary abscess on the HPT axis (Scheitler et al., 2022). *Firmicutes/Bacteroidetes* are often used as quantitative evidence to map the role of gut microbiota in various neurological and psychiatric diseases (Kiseleva et al., 2011). Most of these gut microbiota were participated in vitamin B6, immune system, energy and amino acid metabolism (Wu et al., 2020).

This study employed fecal metabolomics and 16S rRNA gene sequencing technologies to deeply investigate the interaction between the gut microbiota and KYD, as well as the intrinsic regulatory mechanism of CDG on KYD gut microbiota. It was found that CDG could regulate a variety of microorganisms. After RG administration, it regulated 17 bacterial phyla and 9 related metabolites; after WG administration, the gut microbiota of rats underwent significant changes, with an increase of 19 phyla and 12 related metabolites compared to the RG group. CDG mainly exerts therapeutic effects on KYD rats by regulating vitamin B6 metabolism, including pathways such as amino acids, arachidonic acid, linoleic acid, pyrimidine, and steroid biosynthesis metabolism (Zhang X. et al., 2023; Wu et al., 2024). Figure 8C shows that there is a correlation between gut microbiota, fecal metabolites, and HPT axis ELISA indicators. The abundance of pyridoxamine in feces is highly positively correlated with the abundance of *Bifidobacterium*, but negatively correlated with various probiotics. KYD modeling reduced the abundance of various probiotics in rats, but did not significantly inhibit *Bifidobacterium*. *Bifidobacterium* is involved in vitamin B synthesis, thereby increasing the content of pyridoxamine in the gastrointestinal tract (Wu et al., 2023). The change in the abundance of pyridoxamine in feces indicates that it is not a product of KYD rats themselves (Yu et al., 2023), but a result of the disruption of the gut microbiota in KYD rats (Glavan et al., 2023) (Ortiga‐Carvalho et al., 2011). After CDG intervention, both *Bifidobacterium* and pyridoxamine levels were reversed.

As a key member of the gut microbiota, *Bifidobacterium* can synthesize folic acid and affect host metabolism and endocrine functions. The induced folic acid synthesis may affect TRH expression by regulating the HPT axis (Xiang et al., 2021; Carvalho and Dupuy, 2017). Folic acid metabolites in the host can affect neurotransmitter synthesis (D’Aimmo et al., 2023), thereby regulating nervous system functions, including TRH expression of the HPT axis and thyroid hormone levels (Jiang et al., 2022). *Lactobacillus* plays an important role in the transport and metabolism of vitamin B6 (Cheng et al., 2023), affecting the production of T_4_ hormone (Mulligan and Snell, 1977). After RG/WG administration, the fecal metabolite uridine can change the ratio of *Firmicutes* to *Bacteroidetes*, regulate the composition of the gut microbiota, and affect glucose and lipid metabolism by regulating glucose content (Liu et al., 2021).

This study is a preliminary exploratory experimental research, strictly adhering to the “4R principles of ethnopharmacology” in the design and conduct of the research. The experiment utilized omics approaches, integrating 16S rDNA gene sequencing technology, to deeply explore the therapeutic mechanism of CDG in improving KYD by modulating the gut microbiota and endogenous metabolism. During the research, we compared the chemical compositions of RG and WG, comprehensively evaluated their intervention effects on biochemical indicators of plasma, liver, urine, and other tissues, as well as their regulatory effects on the metabolites and gut microbiota of KYD rats. We also deeply investigated the correlation between the gut microbiota and fecal metabolomics. By analyzing the potential endogenous metabolites and microbial genera in KYD rats, we precisely identified the key metabolites and effective bacterial genera regulated by CDG. The results of this study indicate that CDG has the potential to act on the HPT axis, alter the endogenous metabolic pathways of KYD rats, and thereby exert therapeutic effects on KYD by optimizing the gut microbiota, laying the foundation for further in-depth research.

## 5 Conclusion

In this study, a KYD rat model was constructed using propylthiouracil induction. The metabolomic technology of ultra-performance liquid chromatography - tandem mass spectrometry (UPLC-MS/MS) was employed to deeply detect the regulatory effects of CDG on endogenous metabolites in KYD model rats. PCA and OPLS-DA were used to deeply mine and analyze the relevant data, identifying 126 biomarkers involving several key metabolic pathways, including pyrimidine metabolism, GPI-anchored biosynthesis, one-carbon pool of folate, amino acid metabolism, vitamin B6 metabolism, arachidonic acid metabolism, and serine, threonine and glycine metabolism. This study further explored the complex interactions between KYD and the gut microbiota by combining 16S rRNA gene sequencing technology. The total glycosides of *Cistanche deserticola* significantly improved the fecal metabolic characteristics and microbiota diversity of KYD rats, indicating that its therapeutic effects on KYD have positive impacts in multiple aspects. This study not only provides a comprehensive and systematic analysis of the potential mechanisms of CDG in treating KYD but also contributes valuable research results for the development of new KYD treatment strategies.

## Data Availability

The original contributions presented in the study are publicly available. This data can be found here: https://doi.org/10.6084/m9.figshare.29552231.
