# Supplementary material for: Comparative effects of raw and processed cistanche glycosides on the HPT axis and gut microbiota in a rat model of kidney-yang deficiency
Source: Front Pharmacol. 2025 Jul 25;16:1597564. doi: 10.3389/fphar.2025.1597564 (PMC12331631; doi:10.3389/fphar.2025.1597564)
Supplement: Supplementary file 1 [file Supplementaryfile1.docx]

Supplementary Material

# Table S1 Identification results and change trends of important differential metabolite

| No. | Samlpes | Metabolites | RT(s) | *m/z* | Formula | VIP | P | CN *vs* MD | MD *vs* RG | MD *vs* WG |
| --- | --- | --- | --- | --- | --- | --- | --- | --- | --- | --- |
| 1 | feces | L-cis-3-Amino-2-pyrrolidinecarboxylic acid | 483.3340 | 131.0813 | C5H10N2O2 | 1.6534 | 0.0435 | ↓ | ↑ | ↑ |
| 2 | feces | 4,6-Dihydroxyquinoline | 123.8920 | 162.0546 | C9H7NO2 | 1.6459 | 0.0099 | ↓ | ↑ | ↑ |
| 3 | feces | N-Acetyl-D-glucosaminyldiphosphodolichol | 287.9535 | 588.2353 | C28H51NO12P2(C5H8)n | 1.9393 | 0.0063 | ↓ | ↑ | ↑ |
| 4 | feces | (+)-2,3-Dihydro-3-methyl-1H-pyrrole | 283.1595 | 84.0811 | C5H9N | 1.8147 | 0.0278 | ↓ | ↓ | ↓ |
| 5 | feces | Nornicotine | 234.1885 | 149.1069 | C9H12N2 | 1.6103 | 0.0484 | ↑ | ↓ | ↓ |
| 6 | feces | 6-Hydroxy-1H-indole-3-acetamide | 275.3090 | 191.0810 | C10H10N2O2 | 2.1840 | 0.0190 | ↑ | ↓ | ↓ |
| 7 | feces | Isonicotinic acid | 367.3250 | 124.0391 | C6H5NO2 | 1.5121 | 0.0440 | ↓ | ↓ | ↑ |
| 8 | feces | Thyrotropin releasing hormone | 524.6570 | 363.1749 | C16H22N6O4 | 1.0013 | 0.0391 | ↑ | ↓ | ↓ |
| 9 | feces | Isopropylpyrazine | 336.6640 | 123.0915 | C7H10N2 | 2.0963 | 0.0028 | ↑ | ↓ | ↓ |
| 10 | feces | 4-Aminophenol | 49.5752 | 110.0601 | C6H7NO | 1.8114 | 0.0305 | ↑ | ↓ | ↑ |
| 11 | feces | 3-Hydroxymethylantipyrine | 236.2175 | 205.0966 | C11H12N2O2 | 2.4677 | 0.0000 | ↓ | ↑ | ↑ |
| 12 | feces | 4-Trimethylammoniobutanal | 277.5520 | 130.1224 | C7H16NO | 2.0851 | 0.0023 | ↑ | ↓ | ↓ |
| 13 | feces | (+)-threo-2-Amino-3,4-dihydroxybutanoic acid | 94.2430 | 136.0615 | C4H9NO4 | 1.5909 | 0.0395 | ↑ | ↑ | ↑ |
| 14 | feces | Pyridoxamine | 247.5500 | 169.0966 | C8H12N2O2 | 2.3722 | 0.0026 | ↑ | ↓ | ↓ |
| 15 | feces | 3-(3,4-Dihydroxyphenyl)-2-methoxypropionic acid | 292.4740 | 213.0763 | C10H12O5 | 1.6119 | 0.0323 | ↑ | ↓ | ↓ |
| 16 | feces | 2-Methyl-3-(2-methylpropyl)pyrazine | 208.7440 | 151.1226 | C9H14N2 | 2.1939 | 0.0050 | ↑ | ↑ | ↓ |
| 17 | feces | 2,5-Diethylpyrazine | 229.3205 | 137.1069 | C8H12N2 | 2.1468 | 0.0024 | ↑ | ↓ | ↓ |
| 18 | feces | Laccarin | 313.2600 | 195.1123 | C10H14N2O2 | 2.3028 | 0.0004 | ↓ | ↓ | ↓ |
| 19 | feces | 5-Aminopentanal | 402.0440 | 102.0914 | C5H11NO | 1.5138 | 0.0385 | ↑ | ↓ | ↓ |
| 20 | feces | 5-Chloro-2-(3,5-di-tert-butyl-2-hydroxyphenyl)-2H-benzotriazole | 350.1690 | 358.1595 | C20H24ClN3O | 1.9063 | 0.0253 | ↓ | ↓ | ↓ |
| 21 | feces | Benzenepropanenitrile | 35.0197 | 132.0806 | C9H9N | 1.6838 | 0.0259 | ↑ | ↓ | ↓ |
| 22 | feces | Tryptophyl-Alanine | 369.7490 | 276.1333 | C14H17N3O3 | 1.5733 | 0.0416 | ↓ | - | ↓ |
| 23 | feces | Capsaicin | 348.343 | 305.4167 | C18H27NO3 | 2.5814 | 0.0113 | ↓ | ↑ | ↑ |
| 24 | feces | 6-Methylnicotinamide | 348.999 | 151.0862 | C7H8N2O | 1.6721 | 0.0231 | ↑ | ↑ | ↑ |
| 25 | feces | Glycerophosphocholine | 118.0475 | 258.1076 | C8H20NO6P | 1.7290 | 0.0221 | ↓ | ↓ | ↓ |
| 26 | feces | 5-Methoxytryptophan | 374.4185 | 235.1070 | C12H14N2O3 | 1.7452 | 0.0184 | ↑ | ↑ | ↓ |
| 27 | feces | 2-Methoxy-3-(2-methylpropyl)pyrazine | 250.6430 | 167.1174 | C9H14N2O | 2.0855 | 0.0040 | ↑ | ↓ | ↓ |
| 28 | feces | Aldosine | 391.7380 | 255.1330 | C12H18N2O4 | 2.3109 | 0.0006 | ↓ | ↓ | ↑ |
| 29 | feces | N'-Hydroxymethylnorcotinine | 368.6505 | 193.0966 | C10H12N2O2 | 2.1544 | 0.0016 | ↓ | ↓ | ↓ |
| 30 | feces | Tridemorph | 151.0390 | 298.3095 | C19H39NO | 1.5963 | 0.0228 | ↑ | ↓ | ↓ |
| 31 | feces | 2-Ethoxy-5-methylpyrazine | 84.1792 | 139.0863 | C7H10N2O | 2.1784 | 0.0004 | ↑ | ↑ | ↓ |
| 32 | feces | Pyridoxal | 347.8830 | 168.0651 | C8H9NO3 | 1.8237 | 0.0106 | ↑ | ↓ | ↓ |
| 33 | feces | (17alpha,23S)-17,23-Epoxy-29-hydroxy-27-norlanosta-1,8-diene-3,15,24-trione | 314.4310 | 469.2896 | C29H40O5 | 2.1834 | 0.0013 | ↓ | ↓ | ↑ |
| 34 | feces | Genipinic acid | 109.1715 | 243.0868 | C11H14O6 | 1.6754 | 0.0231 | ↑ | ↑ | ↓ |
| 35 | feces | 1-Nitroheptane | 339.7860 | 146.1172 | C7H15NO2 | 1.7095 | 0.0257 | ↑ | - | ↓ |
| 36 | feces | 5'-Hydroxycotinine | 353.6340 | 193.0968 | C10H12N2O2 | 2.4278 | 0.0000 | ↑ | - | ↓ |
| 37 | feces | Spirostane-3,6-dione | 342.0470 | 429.2943 | C27H40O4 | 1.4406 | 0.0314 | ↓ | ↑ | ↑ |
| 38 | feces | Cytokinin B | 364.0680 | 226.1068 | C12H11N5 | 1.6504 | 0.0397 | ↑ | ↓ | ↑ |
| 39 | feces | Calcitriol | 34.9887 | 416.3244 | C27H44O3 | 1.9232 | 0.0422 | ↑ | ↓ | ↓ |
| 40 | feces | Octadecanamide | 153.3125 | 284.2937 | C18H37NO | 1.5150 | 0.0400 | ↓ | ↓ | ↑ |
| 41 | feces | Lysyl-Lysine | 579.8630 | 275.2069 | C12H26N4O3 | 2.1325 | 0.0035 | ↑ | ↓ | ↑ |
| 42 | feces | Nordihydrocapsaicin | 252.2640 | 294.2053 | C17H27NO3 | 1.5949 | 0.0220 | ↑ | ↓ | ↓ |
| 43 | feces | Erinacine D | 181.6390 | 479.2969 | C27H42O7 | 1.6093 | 0.0258 | ↓ | ↓ | ↑ |
| 44 | feces | Avenanthramide E | 355.6385 | 314.1047 | C17H15NO5 | 2.3371 | 0.0001 | ↑ | ↓ | ↓ |
| 45 | feces | 2-Isopropyl-3-methoxypyrazine | 278.7400 | 153.1018 | C8H12N2O | 1.9034 | 0.0101 | ↓ | ↓ | ↓ |
| 46 | feces | Tyrosyl-Valine | 418.3525 | 281.1487 | C14H20N2O4 | 1.9534 | 0.0129 | ↓ | ↑ | ↑ |
| 47 | feces | 2-Acetoxy-3-geranylgeranyl-1,4-dihydroxybenzene | 224.1240 | 441.2939 | C28H40O4 | 1.4048 | 0.0379 | ↓ | ↑ | - |
| 48 | feces | 2,6-Toluenediamine | 261.2850 | 123.0915 | C7H10N2 | 2.0389 | 0.0117 | ↑ | ↓ | ↓ |
| 49 | feces | 2-Isopropyl-3,5-dimethoxy-6-methylpyrazine | 204.5980 | 197.1278 | C10H16N2O2 | 2.2046 | 0.0002 | ↑ | ↓ | ↓ |
| 50 | feces | Validamycin A | 387.1015 | 498.2168 | C20H35NO13 | 2.1568 | 0.0015 | ↓ | ↓ | ↓ |
| 51 | feces | Threoninyl-Phenylalanine | 392.8920 | 267.1329 | C13H18N2O4 | 2.4106 | 0.0001 | ↑ | ↓ | ↓ |
| 52 | feces | Glycyl-Gamma-glutamate | 433.4540 | 204.0974 | C7H13N3O4 | 2.0711 | 0.0263 | ↑ | ↓ | ↓ |
| 53 | feces | Isobutyryl-L-carnitine | 389.4070 | 232.1537 | C11H21NO4 | 1.4952 | 0.0323 | ↓ | ↓ | ↓ |
| 54 | feces | 9,10-epoxyoctadecanoic acid | 33.9600 | 299.2572 | C18H34O3 | 1.7835 | 0.0164 | ↑ | ↓ | ↓ |
| 55 | feces | beta-Vatirenene | 164.6020 | 203.1788 | C15H22 | 1.9462 | 0.0314 | ↑ | ↑ | ↓ |
| 56 | feces | N-a-Acetylcitrulline | 440.6000 | 218.1130 | C8H15N3O4 | 2.2419 | 0.0179 | ↓ | ↓ | ↑ |
| 57 | feces | Fumigaclavine A | 281.0650 | 299.1743 | C18H22N2O2 | 1.7422 | 0.0254 | ↑ | ↓ | ↓ |
| 58 | feces | Sphinganine | 153.3250 | 302.3042 | C18H39NO2 | 1.5279 | 0.0383 | ↓ | ↑ | ↑ |
| 59 | feces | Alanyl-Tyrosine | 389.1780 | 253.1174 | C12H16N2O4 | 2.1623 | 0.0023 | ↑ | ↑ | ↓ |
| 60 | feces | Glutaminylphenylalanine | 232.1110 | 294.1437 | C14H19N3O4 | 2.2237 | 0.0018 | ↑ | ↓ | ↓ |
| 61 | feces | Valproic acid glucuronide | 403.3680 | 321.1545 | C14H24O8 | 1.7886 | 0.0340 | ↓ | ↓ | ↑ |
| 62 | feces | Ercalcitriol | 162.344 | 428.3284 | C28H44O3 | 1.8624 | 0.0245 | ↑ | ↓ | ↓ |
| 63 | feces | Ganoderic acid I | 364.0130 | 533.3069 | C30H44O8 | 1.3987 | 0.0383 | ↑ | ↓ | ↓ |
| 64 | feces | Tanakine | 336.3255 | 220.1326 | C13H17NO2 | 1.4020 | 0.0428 | ↑ | ↑ | ↑ |
| 65 | feces | 3-Oxo-4,6-choladienoic acid | 271.6270 | 371.2567 | C24H34O3 | 1.9381 | 0.0103 | ↓ | ↑ | ↑ |
| 66 | feces | Smilagenone | 60.0978 | 415.3194 | C27H42O3 | 1.5284 | 0.0404 | ↑ | ↓ | ↑ |
| 67 | feces | 3-Hydroxy-4,6-heptadiyne-1-yl 1-glucoside | 91.6964 | 287.1128 | C13H18O7 | 2.2556 | 0.0125 | ↓ | ↑ | ↑ |
| 68 | feces | Dihydrofukinolide | 355.1780 | 393.2230 | C22H32O6 | 2.0314 | 0.0046 | ↑ | ↑ | ↑ |
| 69 | feces | Ampicillin | 470.106 | 350.155878 | C16H19N3O4S | 2.1357 | 0.0087 | ↓ | ↑ | ↑ |
| 70 | feces | Tetracosahexaenoic acid | 162.3395 | 357.2772 | C24H36O2 | 1.7875 | 0.0357 | ↑ | - | ↑ |
| 71 | feces | Unknown 370 | 310.9335 | 413.2994 | C27H40O3 | 1.5158 | 0.0297 | ↓ | ↑ | ↑ |
| 72 | feces | Americine | 156.6980 | 546.3077 | C31H39N5O4 | 1.5537 | 0.0207 | ↑ | ↓ | - |
| 73 | feces | PC(22:5(7Z,10Z,13Z,16Z,19Z)/16:0) | 63.4750 | 808.5779 | C46H82NO8P | 2.1506 | 0.0026 | ↓ | ↑ | ↑ |
| 74 | feces | (R)-Pelletierine | 53.86321 | 142.1223 | C8H15NO | 1.9856 | 0.0099 | ↑ | ↑ | ↓ |
| 75 | feces | 5-Hexyltetrahydro-2-furanoctanoic acid | 47.6551 | 299.2570 | C18H34O3 | 2.0361 | 0.0072 | ↓ | ↑ | ↑ |
| 76 | feces | Desglucocoroloside | 177.0030 | 505.3137 | C29H44O7 | 1.6417 | 0.0129 | ↓ | ↓ | ↓ |
| 77 | feces | Falcarindiol | 164.6025 | 261.1839 | C17H24O2 | 1.4747 | 0.0456 | ↓ | ↑ | ↑ |
| 78 | feces | L,L-Cyclo(leucylprolyl) | 279.9340 | 211.1435 | C11H18N2O2 | 2.3059 | 0.0001 | ↓ | ↑ | ↑ |
| 79 | feces | Asparaginyl-Leucine | 322.4910 | 246.1439 | C10H19N3O4 | 1.5223 | 0.0246 | ↑ | ↓ | ↓ |
| 80 | feces | 4-Phenyl-2-butyl acetate | 163.5030 | 193.1218 | C12H16O2 | 1.7807 | 0.0310 | ↑ | ↓ | ↓ |
| 81 | feces | Polyporusterone C | 60.2682 | 477.3198 | C28H44O6 | 1.8198 | 0.0163 | ↓ | ↑ | ↑ |
| 82 | feces | Alanyl-Valine | 369.7940 | 189.1229 | C8H16N2O3 | 1.5379 | 0.0225 | ↑ | ↑ | ↑ |
| 83 | feces | Flazine | 213.3310 | 309.0857 | C17H12N2O4 | 1.6806 | 0.0482 | ↑ | ↓ | ↓ |
| 84 | feces | Acetylcysteine | 122.7730 | 164.0371 | C5H9NO3S | 1.6020 | 0.0260 | ↓ | ↓ | ↓ |
| 85 | feces | Valyl-Hydroxyproline | 222.5240 | 231.1332 | C10H18N2O4 | 1.9894 | 0.0007 | ↓ | ↑ | ↑ |
| 86 | feces | 2-Methyl-4-phenyl-2-butyl 2-methylpropanoate | 36.1425 | 235.1686 | C15H22O2 | 1.6327 | 0.0256 | ↑ | ↑ | ↑ |
| 87 | feces | Carnosine | 351.3780 | 227.1131 | C9H14N4O3 | 1.8511 | 0.0269 | ↑ | ↓ | ↓ |
| 88 | feces | Folic acid | 203.0630 | 442.1435 | C19H19N7O6 | 1.8214 | 0.0280 | ↑ | ↑ | ↓ |
| 89 | feces | alpha-Amylcinnamyl acetate | 164.6005 | 247.1684 | C16H22O2 | 2.1553 | 0.0024 | ↑ | ↓ | ↑ |
| 90 | feces | Adlupone | 239.638 | 483.3410805 | C31H46O4 | 1.8695 | 0.0134 | ↓ | ↑ | ↑ |
| 91 | feces | 1-Isothiocyanato-7-(methylthio)heptane | 166.8555 | 204.0859717 | C9H17NS2 | 1.9631 | 0.0251 | ↑ | ↑ | ↑ |
| 92 | feces | (2RS,5RS)-(E)-2-(2-Phenylethenyl)-1,3-dioxan-5-ol | 30.5069 | 207.1008602 | C12H14O3 | 2.6321 | 0.0021 | ↑ | ↓ | ↓ |
| 93 | feces | Humulinone | 203.259 | 379.2102588 | C21H30O6 | 2.7537 | 0.0066 | ↓ | ↓ | ↑ |
| 94 | feces | Endomorphin-2 | 423.004 | 572.2896747 | C32H37N5O5 | 2.6521 | 0.0106 | ↑ | ↓ | ↓ |
| 95 | feces | 1,9-Nonanedithiol | 170.8285 | 193.1077935 | C9H20S2 | 1.4252 | 0.0314 | ↑ | ↓ | ↓ |
| 96 | feces | 2,4,6-Trimethyl-4-phenyl-1,3-dioxane | 32.8491 | 207.1372581 | C13H18O2 | 2.8632 | 0.0145 | ↑ | ↑ | ↑ |
| 97 | feces | Simvastatin | 162.336 | 418.259501 | C25H38O5 | 1.4637 | 0.0363 | ↓ | ↑ | ↑ |
| 98 | feces | N-[(5-Hydroxy-2-pyridinyl)methyl]adenosine | 233.331 | 375.1441809 | C16H18N6O5 | 2.5748 | 0.0136 | ↑ | ↓ | ↑ |


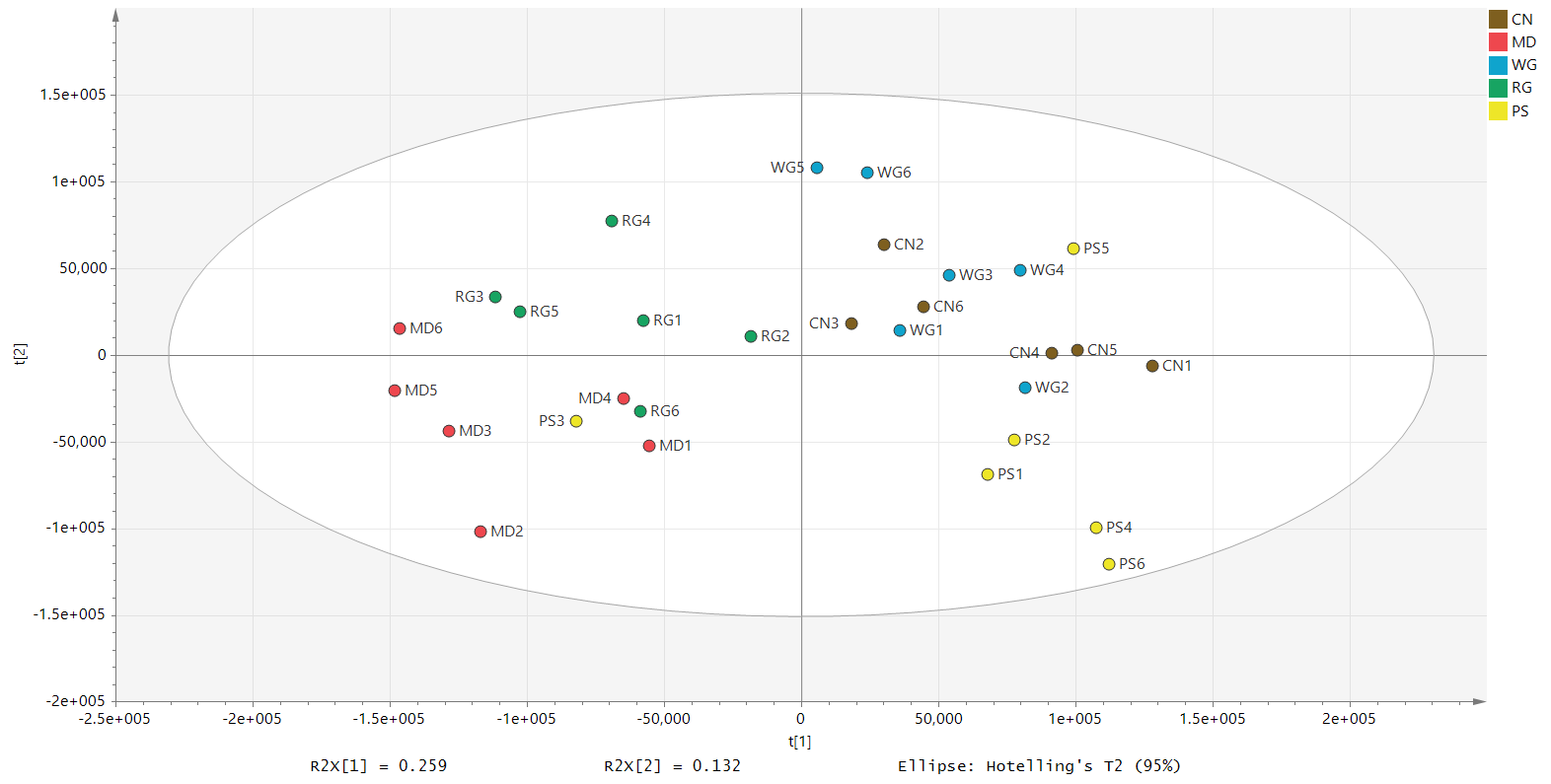


**Figure S1.** Five-group PCA score map based on fecal metabolomics.


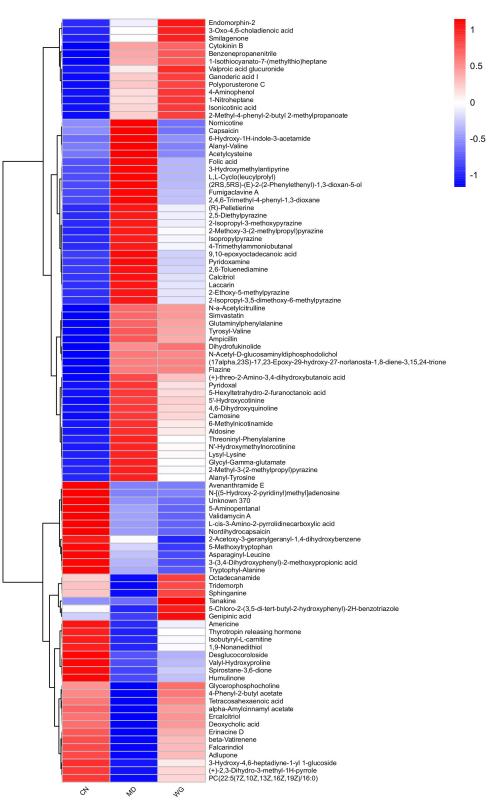

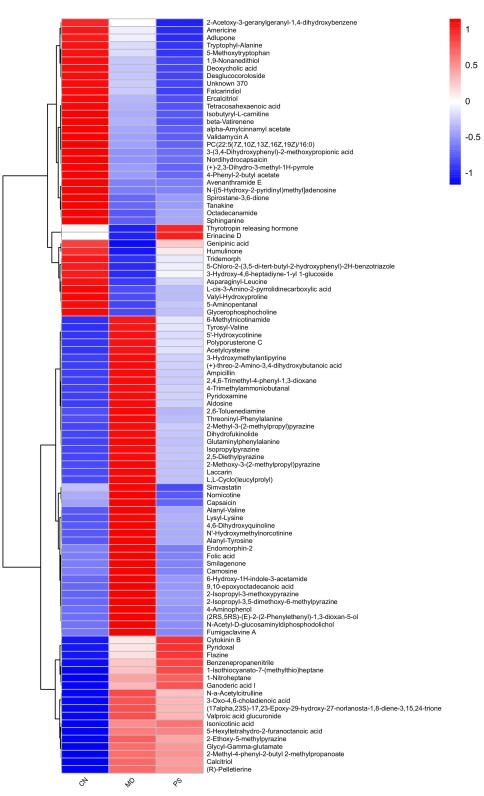

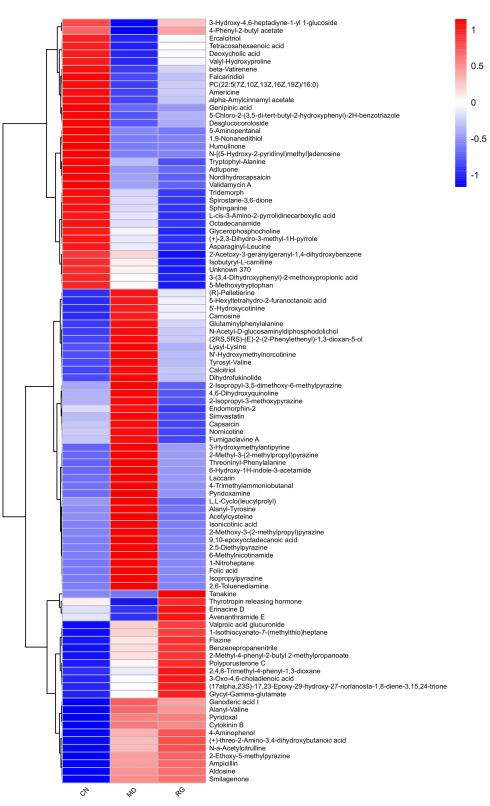


**Figure S2** Heat maps of fecal metabolic difference of RG, WG and PS, with the degree of change marked in red (up-regulation) and blue (down-regulation).


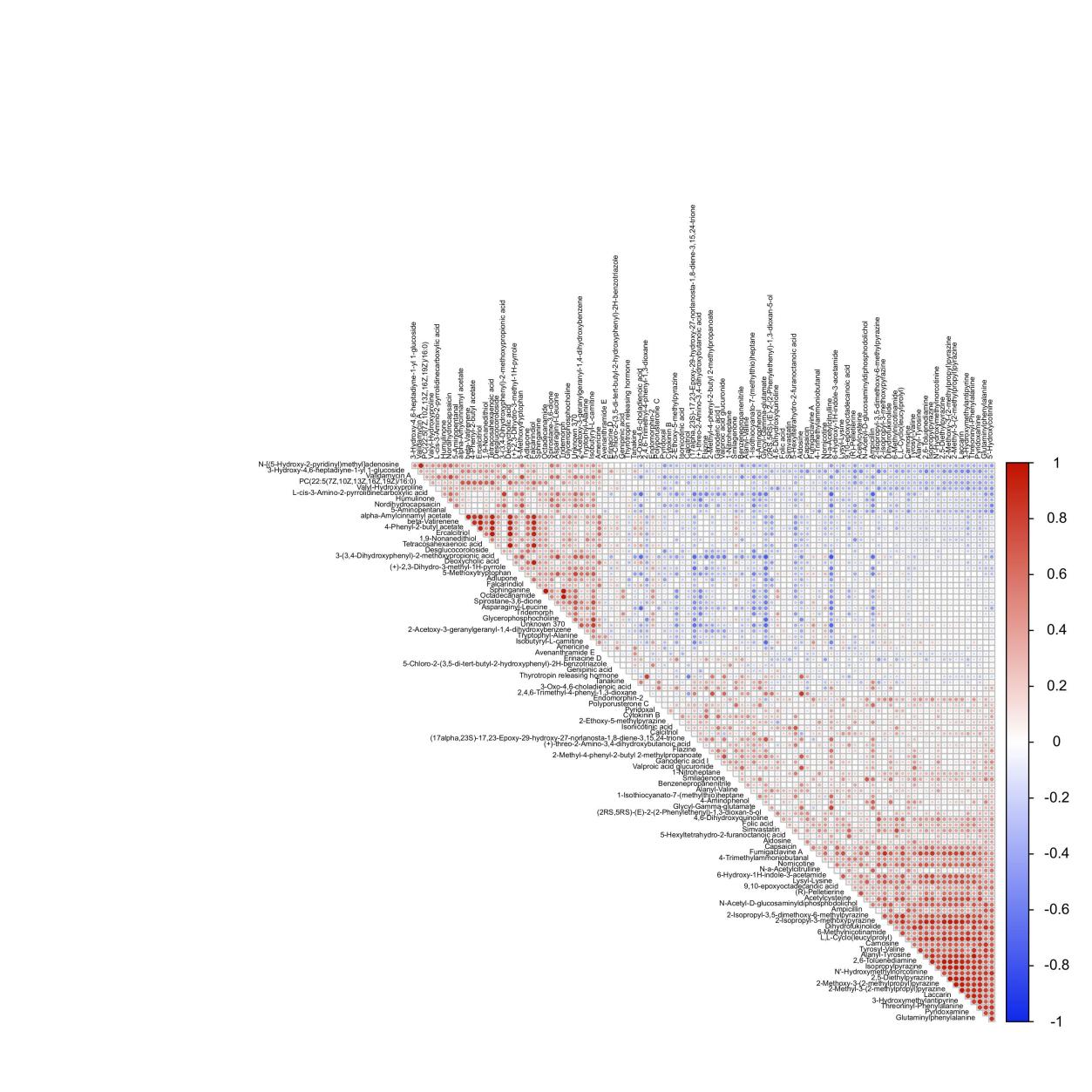


**Figure S3** Correlation analysis heat map of 98 fecal metabolites .

**
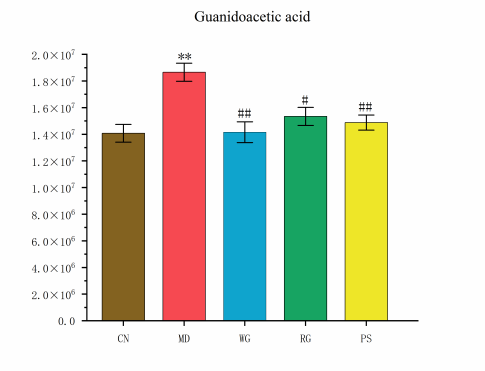

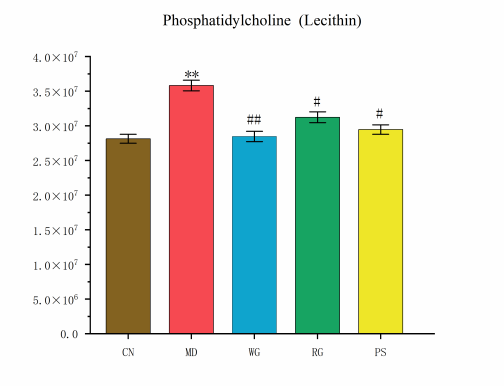

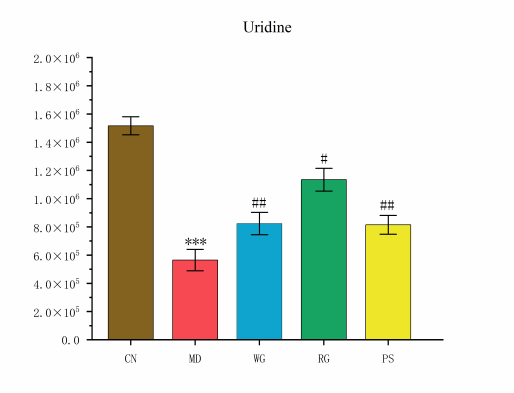
**

**
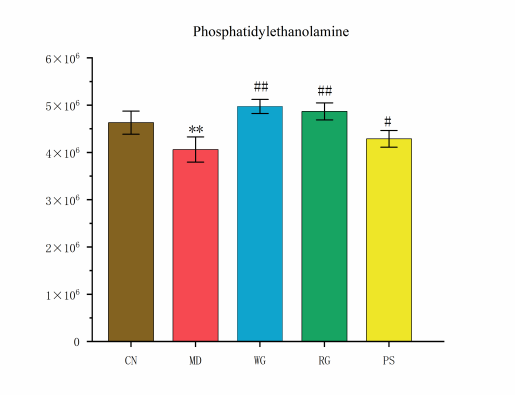

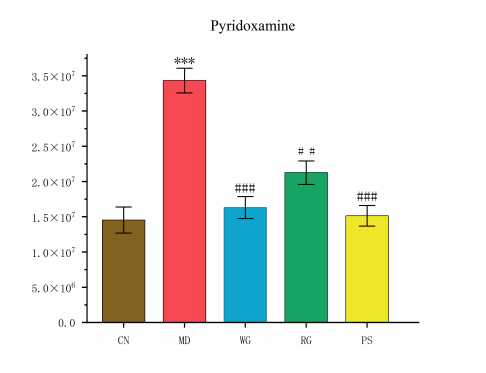

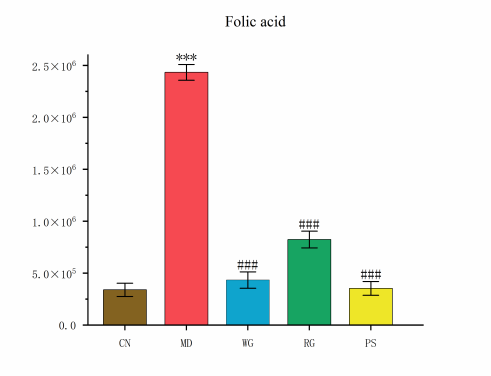
**

**
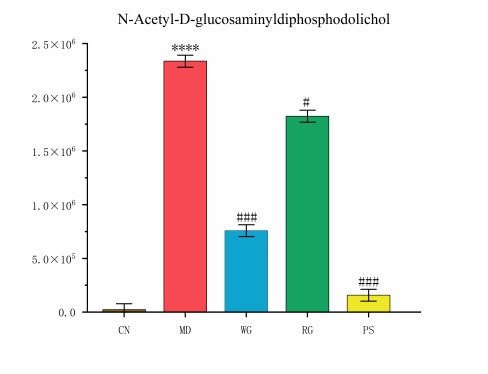
**

**Figure S4.** Representative histogram of CDG improving fecal metabolic disorders in kidney-yang deficiency rats.
